# Properties of Dicationic Disiloxane Ionic Liquids

**DOI:** 10.3390/molecules25122949

**Published:** 2020-06-26

**Authors:** Vladimir G. Krasovskiy, Gennady I. Kapustin, Olga B. Gorbatsevich, Lev M. Glukhov, Elena A. Chernikova, Anatoly A. Koroteev, Leonid M. Kustov

**Affiliations:** 1 N. D. Zelinsky Institute of Organic Chemistry, Russian Academy of Sciences, Leninsky prosp. 47, 119991 Moscow, Russia; gik@ioc.ac.ru (G.I.K.); elektron77@mail.ru (L.M.G.); chernikova_e@mail.ru (E.A.C.); 2 N. S. Enikolopov Institute of Synthetic Polymer Materials, Russian Academy of Sciences, ul. Profsoyuznaya 70, 117393 Moscow, Russia; olborg@list.ru; 3 Moscow Aviation Institute, Volokolamskoe Shosse 4, 125993 Moscow, Russia; chkt@yandex.ru; 4 Chemistry Department, Moscow State University, Leninskie Gory 1, bldg 1., 119992 Moscow, Russia; 5 National University of Science and Technology MISiS, Leninsky prosp. 4, 119991 Moscow, Russia

**Keywords:** dicationic ionic liquids, disiloxane linker, volatility, thermal stability, viscosity, hydrolytic stability

## Abstract

A number of dicationic ionic liquids with a disiloxane linker between imidazolium cations and bis(trifluoromethylsulfonyl)imide anion were synthesized and characterized. Melting points, viscosity, and volatility in a vacuum were measured; the thermal and hydrolytic stability of ionic liquids were also studied. The dependence of the properties on the structure of substituents in the cation of the ionic liquid was demonstrated.

## 1. Introduction

Ionic liquids (ILs), due to their specific properties–high polarity and low saturated vapor pressure (volatility), are widely used in various fields of science and technology. As polar solvents, they are used in organic synthesis [1,2], synthesis of metal and metal oxide nanoparticles [3,4] and metal-organic frameworks (MOF) [5,6]. High thermal stability combined with high polarity determines their use as electrolyte components in lithium batteries and solar cells [7,8]. Low saturated vapor pressure allows the use of ionic liquids as benign solvents for green chemistry [9]. The field of green chemistry also includes the use of ILs for carbon dioxide storage [10] and plastic recycling via depolymerization [11]. High thermal stability and thermal characteristics (heat capacity and heat transfer coefficient) allow them to be used as heat transfer agents in solar energy utilization systems and in heat exchange installations for heating and cooling [12,13,14]. A special feature of ionic liquids is the possibility of using them as a working fluid under dynamic vacuum conditions [15,16,17].

For widespread use of ionic liquids, low viscosity is also required along with the high thermal stability and low volatility. At the same time, the viscosity and volatility of ionic liquids are largely interrelated properties, since both of these parameters are determined by both Coulomb and van der Waals interactions between the ions that make up the specific IL. The presence of Coulomb interaction between cations and anions is the reason for significantly lower volatility of ILs compared to molecular liquids. Dicationic ILs thus differ from other ILs by their much lower volatility compared to “normal” ILs with a single-charged cation, but, and for the same reasons, the dicationic ILs exhibit greater viscosity. The thermal stability of IL is determined by the type of anion and cation that make up their composition, as well as the nature of the functional groups in the cation structure. In particular, the introduction of polar groups usually reduces the thermal stability of an IL. Often, the improvement of some target characteristics of ILs can lead to the deterioration of the other ones. Thus, it is an optimization task to obtain ILs with the specified set of characteristics.

This paper presents the results of a study of the influence of substituents in the imidazolium cation and the structure of the linker between cations on the properties of dicationic ILs with bis(trifluoromethylsulfonyl)imide anion: melting point, viscosity, volatility, thermal, and hydrolytic stability.

## 2. Results and Discussion

### 2.1. Synthesis of ILs

**Scheme 1:**
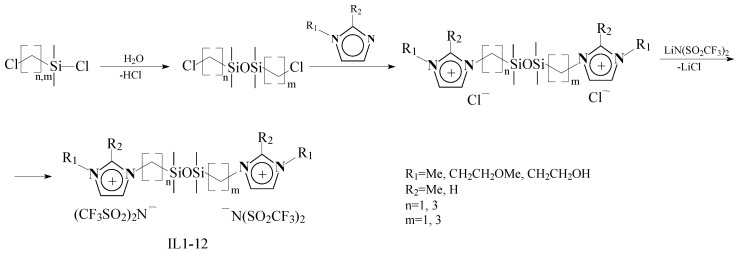
The synthesis of disiloxane dicationic ionic liquids 1–12.

**Figure 1:**
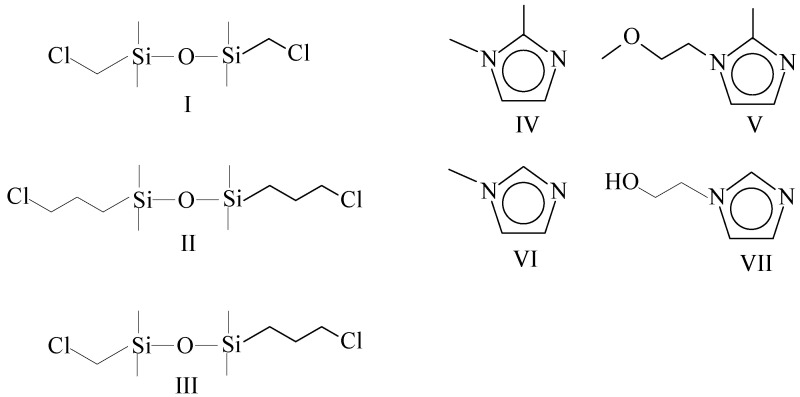
Di(chloroalkyl)tetramethyldisiloxanes and substituted imidazoles used for the synthesis of ionic liquids.

**Scheme 2:**
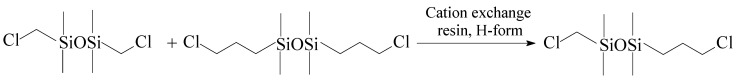
Synthesis of an asymmetric linker–1′,1′,3′,3′-tetramethyl-1′-(chloromethyl)-3′-(3-chloropropyl)disiloxane.

Synthesis of the target dicationic ILs with disiloxane linkers and bis(trifluoromethylsulfonyl)imide (Tf_2_N^-^) anion was performed in three steps (Scheme 1) [18,19]. At the first step, we prepared three different disiloxanes with chloroalkyl groups, which were then used as linkers in the structure of the ILs (Figure 1, structures I-III). The symmetrical disiloxanes (I and II) were obtained by hydrolytic condensation of the corresponding dimethyl(chloroalkyl)chlorosilanes. The asymmetrical disiloxane III was synthesized from disiloxanes I and II (Scheme 2). By quaternization of different substituted imidazoles (Figure 1, structures IV-VII) with di(chloroalkyl)disiloxanes (I-III) in acetonitrile, the chloride precursors of the desired ILs were obtained. The target ILs were synthesized from them by ion exchange with lithium bis(trifluoromethylsulfonyl)imide. All these reactions are well studied, they proceed with high yields, almost without the formation of byproducts.

Asymmetric disiloxane III (1′,1′,3′,3′-tetramethyl-1′-(chloromethyl)-3′-(3-chloropropyl)disiloxane, Figure 1) was synthesized from symmetrical disiloxanes 1 and 2 via disproportionation reaction in the presence of a strongly acidic cation exchange resin (Scheme 2).

**Scheme 3:**
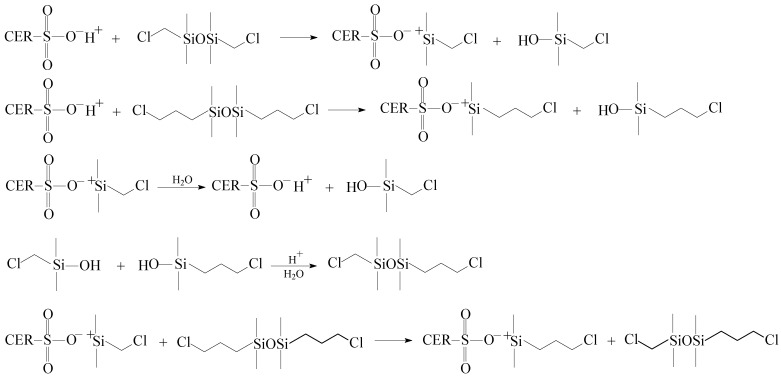
Mechanism of the formation of an asymmetric disiloxane in the presence of a solid strongly acidic cation exchange resin (CER).

The disproportionation reaction of symmetrical disiloxanes proceeds by the mechanism of hydrolytic condensation in the presence of water adsorbed on the surface of strongly acidic cation exchange resin in the H^+^ form (Scheme 3). Purolite CT175 resin, like some other solid catalysts, is most effective when it contains 10 wt % of physically adsorbed water [20,21,22,23].

**Figure 2:**
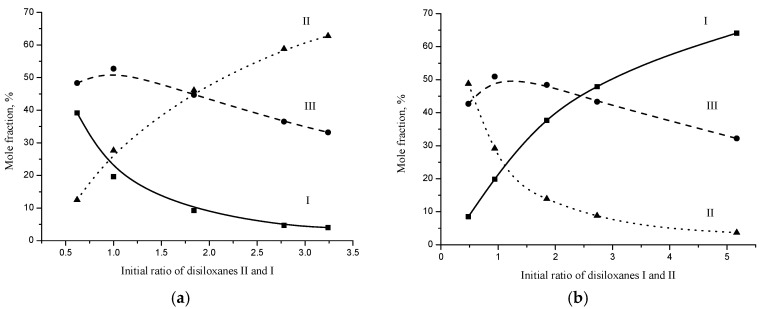
Composition of the reaction mixture (after 6 h at 60 °C) at different initial molar ratios of symmetric disiloxanes (**a**—II/I, **b**—I/II) according to GLC data (numbers I–III correspond to the structures in Figure 1).

The target product—asymmetric disiloxane—is formed as a result of condensation of two different silanols in the bulk of the reaction mixture after splitting of symmetrical disiloxane by the active center of an acid catalyst (Scheme 3). The maximum yield of the asymmetric disiloxane, according to the formal stoichiometry, is achieved with an equimolar ratio of the initial disiloxanes and is equal to 50% (Figure 2). An attempt to achieve the presence of only two products in the equilibrium mixture using a significant excess of one of the disiloxanes was not successful. Even with a 5-fold excess of one of the initial symmetrical disiloxanes, the content of the other symmetrical disiloxane in the equilibrium mixture was below 5% (Figure 2). Therefore, the synthesis of asymmetric disiloxane was carried out at an equimolar ratio of the initial reagents. According to formal kinetics, the composition of the equilibrium mixture of the reaction products at an equimolar quantities of initial disiloxanes must correspond to the ratio [ClCH_2_Si]_2_O:[Cl(CH_2_)_3_Si]_2_O:[ClCH_2_Si]O[Si(CH_2_)_3_Cl] = 1:1:2 = 25%:25%:50%. However, the content of [Cl(CH_2_)_3_Si]_2_O in the reaction mixture is 2–3% higher than the contents of the other components (Figure 2). This is due to the higher activity in the cleavage reaction of the siloxane bond in [ClCH_2_Si]_2_O, in which the induction effect of the chlorine atom makes the siloxane bond more polarized compared to [Cl(CH_2_)_3_Si]_2_O. After a catalyst was removed, the asymmetric disiloxane with T_b_ = 63 °C/1 Torr was isolated by rectification from a mixture of three disiloxanes with a purity of 93%.

The synthesis of ionic liquids with an asymmetric disiloxane linker (ILs 3, 6, 9, and 12, Table 1) was carried out by the same way as with symmetrical disiloxanes (Scheme 1).

Preparation of chloride precursors of ILs 7 and 9 directly by quaternization of 1-(2-hydroxyethyl)imidazole with both siloxanes with methylene spacers proved impossible. The chloride precursors of ILs 1–6 and 10–12 are white crystalline substances soluble in water, while that of IL 8 is a viscous liquid that is also highly soluble in water.

The structures of synthesized bis(trifluoromethylsulfonyl)imide ILs were confirmed by ^1^H, ^13^C-NMR and IR spectroscopy and elemental analysis. The prepared alkylimidazolium ILs contain 60–80 ppm of water, while the IL with hydroxyethyl group has a higher water content of 160 ppm. The properties of ILs are presented in Table 1.

### 2.2. Melting Points

Although ionic liquids are considered to be organic salts with melting (glass transition) points up to 100 °C, their widespread use generally requires their liquid state at room temperature. The introduction of a second ion pair into the structure of a monocationic IL leads to a significant increase in viscosity and an increase in the probability of crystallization. There are two classical ways to reduce the melting point:

1. Increasing the steric effect of a cation and/or anion that prevents crystallization, this can be done by introducing bulk substituents into the cation and by using bulk anions;
2. Violation of the symmetry of the cation.

Both approaches were used to obtain dicationic siloxane ionic liquid with melting points below room temperature (RTILs).

Phase transitions in ionic liquids in the temperature range −100 to +100 °C were studied by differential scanning calorimetry in an inert medium (argon).

On a large number of examples, it is known that ILs with the bis(trifluoromethylsulfonyl)imide anion have very low melting temperatures, compared to the ILs with the same cation, but other anions [24,25]. The large volume of the anion and the presence of trifluoromethyl groups in its structure, which reduce the intermolecular interaction, contribute to reducing the melting temperatures of the ILs.

IL 1 was synthesized by quaternization of 1,2-dimethylimidazole with 1,1,3,3-tetramethyl-1,3-di(chloromethyl)disiloxane (Figure 1, structures I and IV). Under normal conditions, it is a white crystalline solid. According to differential scanning calorimetry (DSC), it melts at 69 °C (Table 1). An increase in the length of the alkyl spacer between the imidazolium cation and the silicon atom from n_C_ = 1 to n_C_ = 3 leads to a decrease in the melting temperature by 10 °C (T_m_ = 59 °C, IL 2, Table 1). Using asymmetric disiloxane as a linker (Figure 1, structure III) also does not allow us to obtain IL with a melting point below room temperature, although this reduces the melting point of IL 3 to 42 °C (Table 1).

ILs 4–6 obtained from 1-methylimidazole, i.e., with no substituents at the position 2 of imidazolium cation, are liquid at room temperature. According to the DSC data, they do not have exact melting temperatures and are characterized only by glass transition temperatures T_g_ = −58...−52 °C (Table 1). The impossibility of crystallization of ILs based on 1-methylimidazole is explained both by the steric effect (higher mobility of imidazolium cations unsubstituted at position 2) and by the specific interaction of the hydrogen atom at position 2 of the imidazolium cation with the Tf_2_N^-^ anion [26,27,28]. The melting point of ILs is also affected by the introduction of polar substituents into the cation [16,17]. Replacing the N-methyl group with a 2-methoxyethyl group in the presence of a methyl group at the position 2 of the imidazolium cation allows us to obtain ILs 10–12 that are viscous liquids at room temperature, characterized by T_g_ = −48...−43 °C (Table 1). IL 8 with an unsubstituted position 2 in the imidazolium cation and a 2-hydroxyethyl group in the position 1 has T_g_ = −53 °C.

Thus, the ILs based on 1,2-dimethylimidazole are most prone to crystallization. The presence of a methyl group at position 2 of the imidazolium cation hindering the intramolecular rotation, increases the probability of intermolecular contacts. This explains the relatively high melting points of these ILs. The introduction of a flexible 2-methoxyethyl group can significantly reduce the melting point. It is interesting to note that the introduction of the 2-hydroxyethyl group into the imidazolium cation, which can form hydrogen bonds with ions and among themselves, does not lead to any increase in the glass transition temperature.

### 2.3. Thermal Stability

Thermal stability is one of the important characteristics of ionic liquids, which is critical for most of their applications. The thermal stability of synthesized ILs was studied by thermogravimetry (TGA) in an inert medium (argon).

The synthesized ionic liquids are characterized by a high thermal stability and, according to TGA data, have decomposition temperatures (T_d_) in the range of 390–440 °C (Table 1). Such high decomposition temperatures are provided by the use of substituted imidazolium cations in the structure of ILs, as the ILs based on this cation are usually distinguished by rather high decomposition temperatures [29]. The stability is also enhanced by the use of the Tf_2_N^-^ anion that is one of the most thermally stable anions used for the synthesis of ILs [30] and siloxane linkers, whose high thermal stability is due to the high stability of the siloxane bond [31,32].

Different lengths alkyl spacers between Si and N atoms, and the introduction of polar substituents in the imidazolium cation have an impact on the thermal stability of the studied ILs. The most thermally stable are ionic liquids 1, 4, and 10 based on 1,2-dimethylimidazole or 1-methylimidazole and containing linkers with methylene spacers. The absence of polar oxygen-containing groups in the cations of these liquids that can form H-bonds and the presence of a short alkyl spacer between the imidazole moiety and the silicon atom causes the enhancement of the thermal stability of ILs by increasing the decomposition temperature to 435–440 °C. The presence of rather mobile hydrogen atom at the position 2 of the imidazolium cation (IL 4) somewhat reduces the thermal stability, but only by 3 °C (Table 1).

The increase in the length of the alkyl spacer in the linker from n_C_ = 1 to n_C_ = 3 in IL 2 and IL 11 reduces the decomposition temperature to 415–420 °C. The simultaneous presence in the structure of a propylene spacer and a mobile hydrogen atom at the position 2 of imidazolium cation (IL 5) has a strong effect on the thermal stability of the IL: the decomposition temperature of IL 5 is reduced by 35 °C compared to that of IL 1 (402 °C, Table 1). Replacing the methyl group in IL 5 with a 2-hydroxyethyl group reduces the decomposition temperature of the ionic liquid (IL 8, Table 1) by another 10°, i.e., to 391 °C.

The decomposition temperature of IL 6 with an unsymmetrical linker occupies an average position between the similar characteristics of symmetrical IL with methylene and propylene spacers. IL 3 and IL 12 with symmetrical linkers are characterized by the decomposition temperature of 420 °C (Table 1). In the case of an asymmetrical linker, the absence of a substituent at position 2 of the imidazolium cation has a weaker effect on the decomposition temperature of the IL–it is reduced only to 410 °C (IL 6, Table 1).

Thus, the use of the most thermally stable structural fragments, i.e., substituted imidazolium cation and Tf_2_N^-^ anion, as well as a siloxane linker in the structures of ILs allows one to obtain dicationic ILs with a high thermal stability up to 440 °C (determined by TGA). The presence of a mobile hydrogen atom at position 2 of the imidazolium cation practically does not affect the thermal stability of the dicationic IL with methylene spacers. However, the simultaneous presence of a propylene spacer and unsubstituted position 2 of imidazolium cation reduces the thermal stability of the IL by 35 °C (from 437 to 402 °C). Due to the presence of a hydroxyl group in the alkyl substituent at the nitrogen atom of the imidazolium cation, an even greater drop in the thermal stability of the IL is observed—the decomposition temperature decreases to 391 °C.

### 2.4. Volatility

One of the specific properties of ionic liquids is their low volatility due to Coulomb interactions between the ions. The classical method for estimating the volatility of ionic liquids is to determine their evaporation enthalpy by various physical and chemical methods: Knudsen effusion mass spectrometry [33,34,35,36,37] or calorimetry [38]. Recently, there have been a significant number of studies where the volatility of ILs was evaluated by thermogravimetry [39,40,41]. However, thermogravimetric studies of evaporation processes carried out in an inert environment or in air are usually complicated by thermal or thermo-oxidative destruction of ILs. Evaporation of monocationic ILs was also studied by the weight method using IL distillation in a vacuum [42,43] under conditions when thermal degradation processes were practically absent.

The introduction of polar substituents (OH groups, for example) into the cation structure or an increase in the number of ion pairs in the structures of ILs leads to a significant decrease in their volatility [16,17]. The volatility of dicationic ILs is by an order of magnitude lower than that of monocationic ones. This generally complicates the use of classical methods of thermogravimetry. In the present work, the volatility of dicationic ILs was measured by a gravimetric method using the McBain balance designed for studying adsorption and desorption processes. This highly sensitive instrument allows one to measure a decrease in the weight of a dicationic IL sample with known surface area at a constant temperature under high dynamic vacuum conditions [44,45,46]. The studies were conducted at temperatures of 150, 190, and 220 °C and a vacuum of 1 × 10^−4^ Torr.

The synthesized dicationic ILs 1–6, 8, and 10–12 practically do not evaporate at a temperature of 150 °C, while their volatility at 190 °C is characterized by values <0.4 mg·h^−1^·cm^−2^ (Table 1). An increase in the evaporation temperature to 220 °C led to an increase in the volatility of the ILs by an order of magnitude. The lowest volatility at this temperature is demonstrated by IL 1 with 1,2-dimethylimidazolium cations and a methylene spacer, whose high melting point confirms a higher intermolecular interaction. Partial or complete replacement of the methylene spacers in the cation structure with propylene ones leads to an increase in volatility of resulting ILs. This can be explained by the steric effect (higher screening of the imidazolium cations in the IL with a longer linker) and, as a result, a decrease in the Coulomb interactions. The decrease in melting point in the row of IL 1 < IL 2 < IL 3 is an indication of this effect.

To assess the effect of polar groups that can form H-bonds on the volatility of dicationic ILs, it is interesting to compare the properties of ILs obtained from 1-(2-hydroxyethyl)imidazole, 1-(2-methoxyethyl)-2-methylimidazole and 1-methylimidazole.

For symmetrical ILs with methylene spacers (ILs 1, 4, and 10), the volatility at 220 °C, depending on the nature of substituents at positions 1 and 2 in the imidazolium cation, follows the order (CH_3_, CH3) < (CH_3_, H) < (CH_3_OCH_2_CH_2_, CH_3_). For symmetrical ILs with propylene spacer (ILs 2, 5, 8, and 11), this series looks different: (CH_3_, CH_3_) < (OH, H) < (CH_3_O, CH_3_) ≤ (CH_3_, H).

Comparison of the data on the mass loss of ILs in a vacuum at 220 °C with thermal degradation temperatures (T_d_) shows a correlation between the volatility values of the studied ionic liquids and their thermal stability. If IL 4 (*n* = m = 1, CH_3_, H) is characterized by T_d_ = 435 °C and volatility at 220 °C in a vacuum of 0.22 mg·h^−1^·cm^−2^, IL 5 in similar conditions has T_d_ = 402 °C and the volatility of 0.95 mg·h^−1^·cm^−2^. Returning to the symmetrical IL with spacers of different lengths, we also observe a correlation of the values of volatility with thermal stability: partial or complete replacement of a methylene spacer with propylene one increases the volatility of the ILs against the background of a decrease in the thermal stability (Table 1).

The introduction of polar substituents into the cation (OH- and CH_3_O-groups), which, according to our ideas, should contribute to reducing the volatility of ILs [16,17], as a result of additional intermolecular interactions with the formation of H-bonds, has a complex effect on the properties of ILs. Such a behavior is explained by the participation of polar groups in thermal decomposition processes, which, unlike the evaporation process, are intensified in the presence of the polar groups. Thus, the introduction of the 2-methoxyethyl group leads to increased observed values of volatility in a vacuum due to the effect of shielding of the cation charge compared to the methyl group, as well as, possibly, a higher contribution of thermal degradation processes (ILs 10–12). For IL 8, the increase in intermolecular interaction due to the formation of H-bonds with the participation of OH groups of the substituent in the cation prevails over the contribution from the cation shielding and the intensification of thermal degradation due to the participation of the polar substituent in the IL. The presence of a specific interaction between the hydrogen atom and the anion should not lead to a decrease in volatility, since the participation of the anion in this interaction reduces its ability to participate in the Coulomb interaction with the cation, but the resulting complex demonstrates a steric effect.

A comparison of the volatility of IL 1 and IL 4 shows that the absence of methyl groups in position 2 of the imidazolium cation leads to an increase in the volatility of IL in a vacuum from 0.04 to 0.22 mg·h^−1^·cm^−2^ at 220 °C. This increase in volatility, despite a decrease in the shielding of the cation charge by only one methyl group, can be associated with both a decrease in the contribution of van der Waals interactions between ions and the manifestation of specific interactions of the anion with the hydrogen atom at position 2 of the imidazolium cation. At the same time, the absence of a substituent at position 2 of the imidazolium cation practically does not affect the thermal stability of the ILs with methylene spacers. The increase in the volatility of ILs 5 and 6 to 0.95 and 2.44 mg·h^−1^·cm^−2^ at 220 °C probably is mainly due to the intensification of decomposition processes in the presence of propylene spacers in the cation and rather mobile 2-H atom in imidazole. The abnormally high observed volatility value of IL 6 is probably due to conformational features of its structure, but this assumption requires additional confirmation.

Thus, with increasing the length of the spacer in the cation (decreasing the charge density of the whole cation of an IL), the presence of polar groups in its structure, as well as unsubstituted position 2 of the imidazolium cations results in an increase in the volatility of dicationic ILs. Since the values of the volatility of ILs at 220 °C are too low to obtain condensate in amounts sufficient to assess the evaporation congruence of the studied ILs, it is not yet possible to draw accurate conclusions about the contributions of the evaporation and decomposition processes in the measured values of volatility. The estimation of the volatility of IL 1, for which the processes of thermal destruction in the study of volatility are least expressed, shows that dicationic ILs are characterized by a very low congruent volatility (<0.1 mg·h^−1^·cm^−2^) under experimental conditions.

### 2.5. Viscosity

The kinematic viscosity of the prepared dicationic ILs was measured using an Ostwald flow viscometer. The Tf_2_N^-^ anion used for the synthesis of dicationic ILs, characterized by a low charge density and the presence of trifluoromethyl groups that reduce the degree of intermolecular interaction, provides significantly lower viscosity values of the obtained ILs and retains their high thermal stability [47].

Ionic liquids 4–6, 8, and 10–12 that do not crystallize under experimental conditions were characterized by viscosity values from 245 to 700 cSt at 30 °C (Table 2). The low viscosity values of ILs 4–6 (240–340 cSt at 30 °C) can be explained by the absence of a substituent at position 2 of the imidazolium cations [48]. The second group of ILs, with the cation structure containing polar substituents (CH_3_O- and OH-groups), is characterized by higher values (550–700 cSt at 30 °C) of the kinematic viscosity. The appearance of additional intermolecular interactions with the formation of H-bonds between polar groups leads to an increase in the viscosity of the ILs.

**Figure 3:**
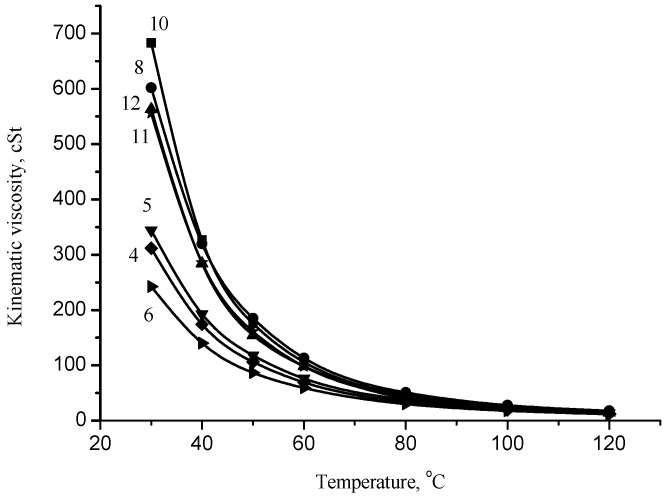
Temperature dependences of kinematic viscosity of IL 4–6, 8, and 10–12.

The temperature dependences of the kinematic viscosity of ILs 4–6, 8, and 10–12 (Figure 3), as well as of most other ionic liquids, are well approximated (R^2^ > 0.99) by the Vogel–Tamman–Fulcher equation [49], which describes the temperature dependences of the viscosity of melts (Table 2).

Thus, the use of the bis(trifluoromethylsulfonyl)imide anion allows us to obtain dicationic disiloxane ILs with rather low viscosity of 240–340 cSt. The viscosity value of the studied ILs is determined by the nature of substituents in positions 1 and 2 of the imidazolium cation (their ability to form hydrogen bonds and thus to increase the intermolecular interaction) and increases in the series (CH_3_,H) < (CH_3_OCH_2_CH_2_,CH_3_) < (HOCH_2_CH_2_,H) (Figure 3). The difference between the viscosities of the studied ILs decreases with a temperature increase. This may be explained by the similarity of the nature of siloxane linkers in all studied compounds, and by the decrease in hydrogen bonding effects at higher temperatures for hydroxyethyl IL.

### 2.6. Hydrolytic Stability

**Scheme 4:**
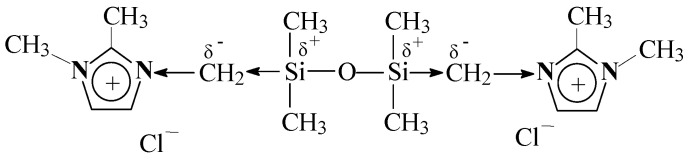
Bonds polarity in the cation of a disiloxane dicationic chloride IL.

A special feature of the synthesized dicationic liquids is the presence of a siloxane linker. Siloxane bonds are distinguished from C-C-bonds by their high mobility and high bond energy. The rotational energy barriers for Si-O and C-C bonds are 0.8 and 11.3 kJ/mol, respectively, and those for Si-CH_3_ and C-CH_3_ bonds are 6.7 and 15.1 kJ/mol, respectively [50]. The dissociation energy of the Si-O bond in hexamethyldisiloxane is 549 kJ/mol, and that for the C-C bond in ethane is 334 kJ/mol [51]. In addition, the siloxane bond is polar, and its ionicity reaches 50% [52,53,54]. The partially ionic nature character of the siloxane bond causes its hydrolysis in certain (rather severe) conditions [55,56,57,58]. The presence of two imidazolium cations in the IL structure leads to additional polarization of Si-C-and Si-O-Si-bonds in the linker and to a decrease in their hydrolytic stability (Scheme 4).

**Scheme 5:**
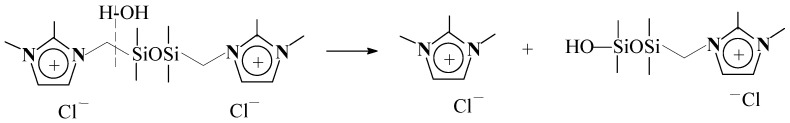
Hydrolysis of the Si-C-bond in the cation of a disiloxane dicationic IL.

This is confirmed, for example, by the formation of a 1–2% of 1,2,3-trimethylimidazolium chloride as the byproduct in the quaternization reaction in the synthesis of ILs 1 and 3. This fact is explained by the presence of traces of water in the reaction mixture due to the high hygroscopicity of substituted imidazoles (Scheme 5).

**Scheme 6:**
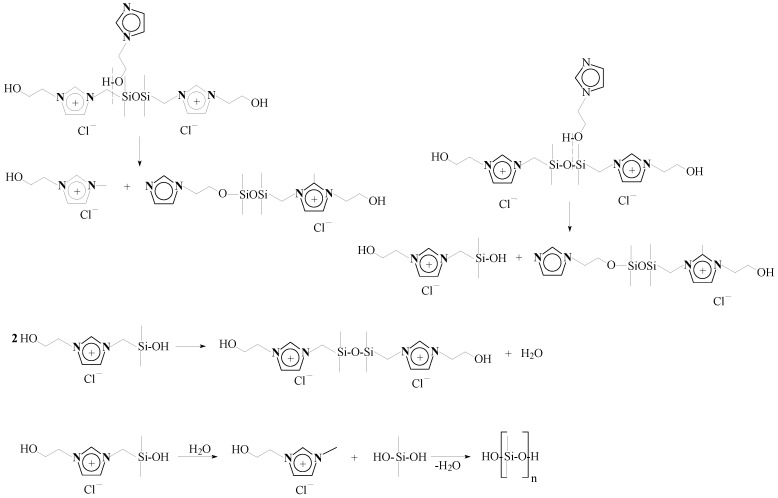
Protolysis side-reactions leading to impossibility of direct synthesis of ILs 7 and 9.

The reason for the impossibility to directly synthesize chloride precursors of ILs 7 and 9 is the presence of a hydroxyl group in 1-(2-hydroxyethyl)imidazole. In the quaternization reaction (acetonitrile, 80 °C), not only Si-C, but also SiOSi-siloxane bond in the linker undergoes protolysis under the action of OH groups in the reagent (Scheme 6).

**Figure 4:**
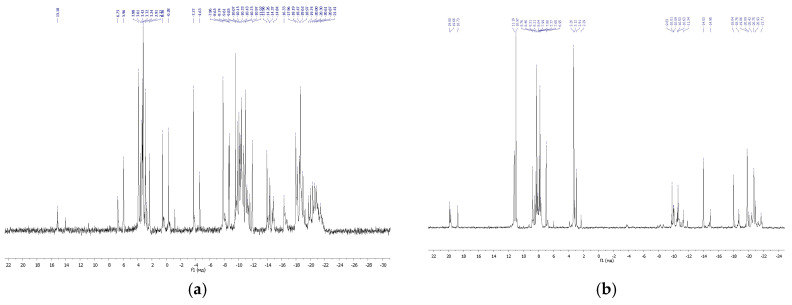

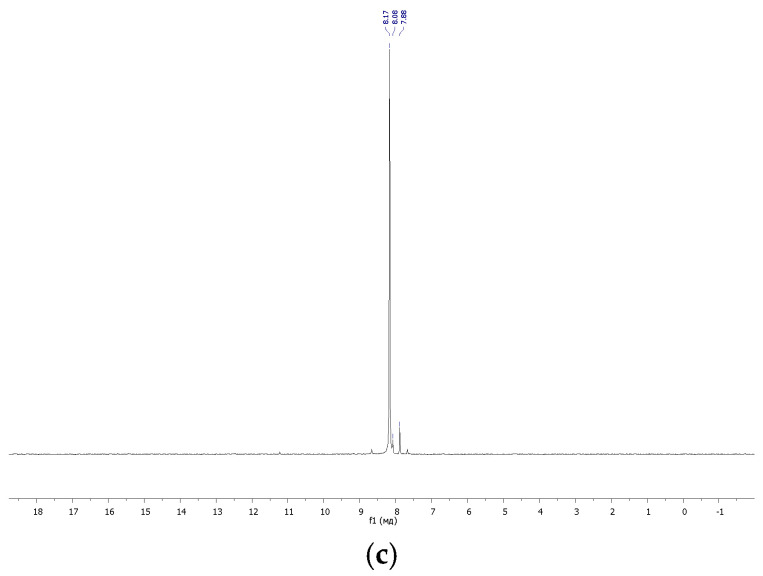
^29^Si NMR spectra of chloride IL 7 (**a**), IL 9 (**b**), and IL 8 (**c**).

The cleavage of the siloxane bond is confirmed by ^29^Si NMR spectroscopy data (Figure 4a,b). A set of peaks in the range of +15 to −23 ppm in the NMR spectra of the products obtained in attempt to directly synthesize the chloride precursors of ILs 7 and 9 indicates the presence not only of various silanols, alkoxysilanes, and disiloxanes, but also siloxane oligomers [53,59]. Confirmation of the influence of the induction effect of cations on the hydrolytic stability of the organosilicon linker is a significantly smaller number of peaks in the NMR spectrum of reaction products when trying to directly synthesize chloride IL 9 with an asymmetric linker (Figure 4b). However, when siloxane precursor with 3-chloropropyl groups was used instead, the quaternization reaction with 2-hydroxyethylimidazole proceeds with formation of only the stable target product (chloride precursor of IL 8). A single peak at 8.17 ppm, corresponding to silicon atoms in a disiloxane linker with propylene spacers, is characteristic of the ^29^Si NMR spectrum of the reaction product (Figure 4c).

**Scheme 7:**
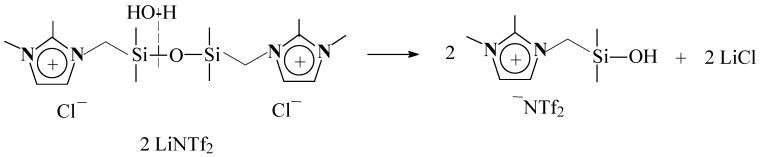
Silanol formation during hydrolysis of a siloxane linker in the IL cation.

The hydrolytic instability of the siloxane linker with methylene spacers is also an issue in an attempt to perform the third stage of synthesis of bis(trifluoromethylsulfonyl)imide IL, i.e., the anion exchange in water. Ion exchange in water (the most commonly used method) involving, for example, the chloride precursor of IL 1 leads to formation of up to 50% of silanol (Scheme 7), which is obtained as a result of hydrolysis of the siloxane bond. Formation of 1,2,3-trimethylimidazolium bis(trifluoromethylsulfonyl)imide was not observed. Apparently, a fluorine-containing anion with pronounced hydrophobic properties prevents the hydrolysis of the Si-C bond during anion exchange in water.

In this regard, the better method is to perform the ion exchange reaction with lithium bis(trifluoromethylsulfonyl)imide in acetonitrile. The most part of lithium chloride formed as a byproduct can be removed by filtration. After this procedure, the residual quantities of lithium chloride and excess of lithium bis(trifluoromethylsulfonyl)imide can be safely removed by conventional water extraction method in water-dichloromethane system. The hydrolytic stability of the target bis(trifluoromethylsulfonyl)imide ILs can be explained both by the hydrophobicity and by the lower nucleophilicity of the bis(trifluoromethylsulfonyl)imide-anion compared to the chloride ion.

Thus, we have reported the use of disiloxane linker in the synthesis of dicationic ionic liquids with methylene spacers between Si atom and imidazolium cation leads to a decrease in the hydrolytic stability of ILs due to an increase in the polarity of Si-C and Si-O-Si bonds due to the induction effect of imidazolium cations. The use of a propylene spacer allows us to eliminate this disadvantage and to obtain hydrolytically stable dicationic disiloxane ILs.

## 3. Materials and Methods

### 3.1. General Information

^1^H, ^13^C, and ^29^Si NMR spectra were measured using a Bruker AM-300 NMR spectrometer. IR spectra were measured using a Nicolet iS50 Fourier-transform infrared spectrometer with an integrated ATR unit (a diamond prism, resolution 4 cm^−1^, number of scans --32). The water content in the ILs was determined using Mettler-Toledo C30 coulometric Karl Fischer titrator using a Hydranal methanol-based reagent. The sample volume was 2 mL. Thermogravimetric analysis was performed using a Derivatograph-C instrument (MOM, Hungary) in an argon atmosphere at a heating rate of 10 K min^−1^ (sample weight, 20 mg). Glass transition temperatures were determined by the DSC method using a DSC-822e differential scanning calorimeter (Mettler-Toledo, Switzerland) in the temperature range from −100 to 100 °C at a sample heating rate of 10 K min^−1^ in an argon atmosphere. The purity of the prepared di(chloroalkyl)disiloxanes and the composition of the reaction mixtures in disproportionation reactions was studied by GC analysis using Chromatec Crystal-5000 chromatograph (Chromatec, Russia) with a 2 m × 3 mm packed column (5% SE-30 on Chromaton-N-AW). The injector temperature was 350 °C, the temperature of the thermal conductivity detector was 310 °C, and the helium flow rate was 30 mL/min. The analysis was performed in the temperature programmed mode (temperature range 150–300° C, heating rate 20 °C/min). The kinematic viscosity of ILs was measured using an Ostwald viscometer with a capillary diameter of 0.8 mm. The viscometer was calibrated at 25 °C using ethylene glycol (Aldrich, 99.8%, water content <0.01%) as the reference liquid. The viscosity of ethylene glycol was checked using the Ubbelohde viscometer calibrated with double distilled water. The volatility of ILs in a vacuum was evaluated using McBain quartz spiral balances. The sample (0.2 g) was placed in a quartz cup fixed on the movable end of the quartz spring of the balances. The surface area of the liquid was 1.3–1.7 cm^2^. The cell with spring and sample cup was placed in a thermostatted aluminum block. The spring extension was determined by measuring the position of the reference marks using a KM-8 cathetometer with an accuracy of 0.02 mm. The spiral used had a sensitivity of 0.3709 mm/mg at 220 °C, and was calibrated at different temperatures with a quartz sample. The setup was evacuated using a diffusion pump. Before measurements at higher temperatures, the samples were dried at 100 °C to a constant weight (15 h) directly in the setup in a vacuum no worse than 10^−4^ Torr.

Dimethyl(chloromethyl)chlorosilane (98%), dimethyl(3-chloropropyl)chlorosilane (97%), 1-methylimidazole (99%), 2-methylimidazole (99%), 1,2-dimethylimidazole (98%), 1-(2-hydroxyethyl)imidazole (97%), 1-chloro-2-methoxyethane (98%), and lithium bis(trifluoromethylsulfonyl)imide (99%) were purchased from Acros and Sigma-Aldrich (St. Louis, MO, USA).

Imidazole derivatives were dried over solid KOH (1-methylimidazole and 1,2-dimethylimidazole) or by azeotropic distillation of absolute acetonitrile. All organic solvents used in the synthesis were previously dried over CaH_2_ and distilled (see Supplementary Materials).

### 3.2. Synthesis of ILs with a Symmetrical Linker (1, 2, 4, 5, 10, and 11)

Methods for the synthesis of symmetrical disiloxane dicationic ILs 1, 4, and 10 with a methylene spacer are described in [19] and with a propylene spacer (ILs 2, 5, and 11) in [18]. The synthesis of 1-(2-methoxyethyl)-2-methylimidazole is described in [19].

*1′,1′,3′,3′-Tetramethyl-1′,3′-bis([1,2-dimethylimidazolium-3-yl]methyl)disiloxane bis(trifluoromethylsulfonyl)imide* (**1**). The yield of IL 1 was 93%. White solid. ^1^H NMR (DMSO-d_6_, 300.13 MHz) δ 0.16 (s, 12H), 2.52 s (s, 6H), 3.75 (s 6H), 3.82 (s, 4H), 7.41 (m, 2H), 7.62 (m, 2H). ^13^C NMR (DMSO-d_6_, 75.47 MHz) δ 0.48, 9.74, 35.26, 40.21, 113.05 * (q *, *J*_F_ = 321.4 Hz), 117.80 *, 121.83, 122.06 *, 122.81, 126.82 *, 143.97. IRS (ATR) 3184, 3151, 3088, 3049, 2965, 2917, 2853, 1588, 1539, 1515, 1418, 1350,1332, 1326, 1265, 1227, 1176, 1132, 1048, 1035, 841, 823, 792, 763, 740, 714, 693, 664, 655, 613, 599, 580, 569, 508, 469, 435, 406. Gross formula: C_20_H_32_N_6_O_9_F_12_S_4_Si_2_, molecular weight 912.92. Calculated, %: C 26.31, H 3.53, N 9.21, F 24.97, S 14.05, Si 6.15. Found, %: C 26.23, H 3.63, N 9.16, F 24.94, S 13.91, Si 6.26.

*1′,1′,3′,3′-Tetramethyl-1′,3′-bis(3-[1,2-dimethylimidazolium-3-yl]propyl)disiloxane bis(trifluoromethylsulfonyl)imide* (**2**). The yield of IL 2 was 95%. White solid. ^1^H NMR (DMSO-d_6_, 300.13 MHz) δ 0.06, (s, 12H), 0.48 (m, 4H), 1.68 (m, 4H), 2.57 (s, 6H), 3.75 (s, 6H), 4.10 (t, *J* = 7.32 Hz, 4H), 7.62 (m, 4H). ^13^C NMR (DMSO-d_6_, 75.47 MHz) δ 0.48, 9.53, 14.54, 23.93, 35.05, 50.50, 113.54 * (q *, *J*_F_ = 321.9 Hz), 117.80 *, 121.21, 122.06 *, 122.75, 126.24 *, 144.61. IRS (ATR) 3186, 3150, 3093, 3053, 2958, 2901, 2810, 1590, 1540, 1467, 1421, 1347, 1330, 1255, 1226, 1176, 1134, 1051, 918, 877, 840, 788, 762, 739, 654, 613, 599, 569, 510, 406. Gross formula: C_24_H_40_F_12_N_6_O_9_S_4_Si_2_, molecular weight 969.03. Calculated: C 29.75, H 4.16, N 8.67, F 23.53, S 13.23, Si 5.80. Found: C 29.66, H 4.27, N 8.60, F 23.50, S 13.19, Si 5.87.

*1′,1′,3′,3′-Tetramethyl-1′,3′-bis([1-methylimidazolium-3-yl]methyl)disiloxane bis(trifluoromethylsulfonyl)imide* (**4**). The yield of IL 4 was 88%. Colorless liquid. ^1^H NMR (DMSO-d_6_, 300.13 MHz) δ 0.16 (s, 12H), 3.87 (s, 6H), 3.89 (s, 4H), 7.52 (m, 2H), 7.69 (m, 2H), 8.90 (s, 2H). ^13^C NMR (DMSO-d_6_, 75.47 MHz) δ −0.96, 36.16, 41.37, 113.52 *(q *, J_F_ = 321.8 Hz), 117.81 *, 122.07 *, 123.54, 124.15, 126.25*, 136.25. IRS (ATR) 3155, 3119, 3100, 2961, 2925, 2853, 1570, 1427, 1347, 1329, 1264, 1226, 1176, 1133, 1050, 1021, 840, 806, 789, 761, 739, 647, 611, 599, 569, 509, 406. Gross formula: C_18_H_28_N_6_O_9_F_12_S_4_Si_2_, molecular weight 884.87. Calculated, %: C 24.43, H 3.20, N 9.50, F 25.76, S 14.49, Si 6.35. Found, %: C 24.33, H 3.35, N 9.46, F 25.73, S 13.51, Si 6.45.

*1′,1′,3′,3′-Tetramethyl-1′,3′-bis([1-methylimidazolium-3-yl]propyl)disiloxane bis(trifluoromethylsulfonyl)imide* (**5**). The yield of IL 5 was 96%. Colorless liquid. ^1^H NMR (DMSO-d_6_, 300.13 MHz) δ 0.06 (s, 12H), 0.42 (m, 4H), 1.78 (m, 4H), 3.86 (s, 6H), 4.13 (t, *J* = 7.2, 4H), 7.69 (m, 2H), 7.73 (m, 2H), 9.08 (s, 2H). ^13^C NMR (DMSO-d_6_, 75.47 MHz) δ −0.01, 14.06, 23.79, 35.66, 51.41, 113.08 * (q *, *J*_F_ = 321.4), 117.34 *, 121.60 *, 122.12, 123.69, 125.87 *, 136.40. IRS (ATR) 3156, 3120, 3102, 2958, 2900, 2808, 1573, 1466, 1431, 1348, 1330, 1256, 1226, 1178, 1133, 1051, 915, 839, 788, 762, 740, 714, 653, 613, 599, 569, 510, 459, 406. Gross formula: C_22_H_36_F_12_N_6_O_9_S_4_Si_2_, molecular weight 940.98. Calculated: C 28.08, H 3.86, N 8.93, F 24.23, S 13.63, Si 5.97. Found: C 27.99, H 3.95, N 8.90, F 24.20, S 13.51, Si 6.07.

*1′,1′,3′,3′-Tetramethyl-1′,3′-bis([1-(2-methoxyethyl)-2-methylimidazolium-3-yl]methyl)disiloxane bis(trifluoromethylsulfonyl)imide* (**10**). The yield of IL 10 was 95%. Light yellow liquid. ^1^H NMR (DMSO-d_6_, 300.13 MHz) δ 0.14 (s, 12H), 2.59 (s, 6H), 3.24 (s, 6H), 3.65 (t, J = 4.9 Hz, 4H), 3.94 (s, 4H), 4.37 (t, J = 5.0 Hz, 4H), 7.61 (m, 2H), 7.75 (m, 2H). ^13^C NMR (DMSO-d_6_, 75.47 MHz) δ −0.80, 9.89, 40.23, 48.09, 58.53, 70.39, 113.55 * (q *, J_F_ = 321.9 Hz), 117.81 *, 122.06, 122.08 *, 122.12, 126.34*, 144.04. IRS (ATR) 3182, 3127, 3087, 2959, 2900, 2840, 2821, 1583, 1531, 1436, 1347, 1329, 1258, 1225, 1178, 1133, 1102, 1050, 1014, 917, 838, 789, 761, 739, 672, 653, 612, 599, 510, 406. Gross formula: C_24_H_40_F_12_N_6_O_11_S_4_Si_2_, molecular weight 1001.03. Calculated: C 28.80, H 4.03, N 8.39, F 22.78, S 12.81, Si 5.61. Found: C 28.69, H 4.10, N 8.32, F 22.76, S 12.77, Si 5.66.

*1′,1′,3′,3′-Tetramethyl-1′,3′-bis(3-[1-(2-methoxyethyl)-2-methylimidazolium-3-yl]propyl)disiloxane bis(trifluoromethylsulfonyl)imide* (**11**). The yield of IL 11 was 94%. Light yellow liquid. ^1^H NMR (DMSO-d_6_, 300.13 MHz) δ 0.05 (s, 12H), 0.45 (m, 4H), 1.71 (m, 4H), 2.61 (s, 6H), 3.25 (s, 6H), 3.66 (t, J = 4.87 Hz, 4H), 4.09 (t, J = 7.24 HZ, 4H), 4.32 (t, J = 5.06 Hz, 4H), 7.65 (m, 4H). ^13^C NMR (DMSO-d_6_, 75.47 MHz) δ -0.04, 9.30, 14.04, 23.39, 47.46, 50.05, 58.14, 68.84, 113.09 * (q *, J_F_ = 321.3 Hz), 117.36 *, 121.10 *, 121.60 *, 121.63, 129.89, 144.25. IRS (ATR) 3183, 3147, 3087, 2955, 2901, 2840, 2822, 1587, 1532, 1452, 1348, 1330, 1255, 1225, 1178, 1134, 1102, 1051, 1014, 917, 877, 838, 788, 762, 739, 714, 672, 653, 613, 599, 569, 511, 406. Gross formula: C_28_H_48_F_12_N_6_O_11_S_4_Si_2_, molecular weight 1057.14. Calculated: C 31.81, H 4.58, N 7.95, F 21.57, S 12.13, Si 5.31. Found: C 28.67, H 4.66, N 7.88, F 21.62, S 12.02, Si 5.40.

### 3.3. Synthesis of ILs with an Asymmetric Linker (3, 6, and 12)

#### General Method

The first stage is the synthesis of an asymmetric linker.

A flask was loaded with 1.6 g (0.007 mol) of 1,1,3,3-tetramethyl-1,3-di(chloromethyl)disiloxane, 2.0 g (0.007 mol) of 1,1,3,3-tetramethyl-1,3-bis(3-chloropropyl)disiloxane, and 0.11 g (3% of the reaction mixture) of Purolite CT 175/2429 cation exchange resin. The reaction mixture was vigorously stirred on a magnetic stirrer at 60 °C for 6 h. The catalyst was removed by filtration. Asymmetric disiloxane with T_b_ = 63 °C/1 Torr was isolated by vacuum distillation from a mixture of three disiloxanes with a purity of 93% (according to GLC). Yield 1.4 g (39%).

The second stage is the synthesis of chloride precursors of ILs 3, 6, and 12.

Substituted imidazole (1,2-dimethylimidazole, 1-methylimidazole, 1-(2-methoxyethyl)-2-methylimidazole) was quaternized with 1′,1′,3′,3′-tetramethyl-1′-(chloromethyl)-3′-(3-chloropropyl)disiloxane in acetonitrile (50% solution) at an equimolar ratio of the initial reagents for 72 h at the boiling point of the solvent. After crystallization, the solid product was collected by filtration and dried in a vacuum.

The third stage is the synthesis of bis(trifluoromethylsulfonyl)imide ILs 3, 6, and 12.

A mixture of chloride precursor and a 30% solution of lithium bis(trifluoromethylsulfonyl)imide (10% excess) in acetonitrile was stirred for 90 min. The LiCl precipitate was removed by filtration, acetonitrile was distilled off in a vacuum, and the residue was dissolved in dichloromethane. The solution was extracted with small portions of water until negative reaction of the wash water with AgNO_3_. The ionic liquid was dried by azeotropic distillation with absolute dichloromethane (100 mL of CH_2_Cl_2_ per 1 g of IL) and then in a vacuum (~0.01 Torr) at room temperature and then at 80 °C.

*1′,1′,3′,3′-Tetramethyl-1′-([1,2-dimethylimidazolium-3-yl]methyl)-3′-(3-[1,2-dimethylimidazolium-3-yl]propyl)disiloxane bis(trifluoromethylsulfonyl)imide* (**3**).The yield of IL 3 was 85%. White solid. ^1^H NMR (300 MHz, DMSO-d_6_) δ 0.05 (s, 6H), 0.17 (s, 6H), 0.49 (m, 2H), 1.67 (m, 2H), 2.53 (s, 3H), 2.58 (s, 3H), 3.76 (s, 6H), 3.80 (s, 2H), 4.07 (t, J = 7.3 Hz, 2H), 7.41 (m, 1H), 7.62 (m, 1H + 2H). ^13^C NMR (75.47 MHz, DMSO-d_6_) δ −0.51, 0.09, 9.45, 9.61, 14.31, 23.75, 34.99, 35.13, 40.45, 50.48, 113.55 * (q *, J_F_ = 321.4 Hz), 117.81 *, 121.18, 121.81, 122.08 *, 122.64, 122.74, 126.34 *, 143.86, 144.58. ^29^Si NMR (59.60 MHz, DMSO-d_6_) δ 3.93, 11.19. IRS (ATR) 3188, 3156, 3094, 3047, 2962, 2938, 2881, 1589, 1538, 1464, 1421, 1349, 1331, 1259, 1237, 1214, 1174, 1138, 1051, 1036, 916, 873, 847, 808, 788, 762, 740, 683, 668, 608, 569, 513, 472, 409. Gross formula C_22_H_36_F_12_N_6_O_9_S_4_Si_2_, molecular weight 940.98. Calculated (%): C, 28.08; H, 3.86; N, 8.93; F, 24.23; S, 13.63; Si, 5.97. Found (%): C, 27.99; H, 3.94; N, 8.89; F, 24.20; S, 13.67; Si, 6.02.

*1′,1′,3′,3′-Tetramethyl-1′-([1-methylimidazolium-3-yl]methyl)-3′-(3-[1-methylimidazolium-3-yl]propyl)disiloxane bis(trifluoromethylsulfonyl)imide* (**6**). The yield of IL 6 was 65%. Light yellow liquid. ^1^H NMR (DMSO-d_6_, 300.13 MHz) δ 0.05 (s, 6H), 0.17 (s, 6H), 0.45 (m, 2H), 1.74 (m, 2H), 3.86 (m, 6H + 2H), 4.12 (t, J = 7.1 Hz, 2H), 7.51 (m, 1H), 7.68 (m, 1H + 1H), 7.73 (m, 1H), 8.91 (s, 1H), 9.08 (s, 1H). ^13^C NMR (DMSO-d_6_, 75.47 MHz) δ -1.2, -0.30, 13.80, 23.61, 35.63, 35.65, 41.08, 51.37, 113.07 * (q *, J_F_ = 321.2 Hz), 117.35 *, 121.61 *, 122.13, 123.09, 123.57, 123.60, 125.87 *, 135.78, 136.47. IRS (ATR) 3155, 3119, 3101, 2960, 2903, 1569, 1431, 1348, 1330, 1260, 1226, 1177, 1133, 1050, 917, 839, 802, 789, 762, 739, 652, 612, 599, 569, 510, 406. Gross formula: C_20_H_32_N_6_O_9_F_12_S_4_Si_2_, molecular weight 912.92. Calculated, %: C 26.31, H 3.53, N 9.21, F 24.97, S 14.05; Si 6.15. Found, %: C 26.24, H 3.69, N 9.17, F 24.93, S 13.93, Si 6.21.

*1′,1′,3′,3′-Tetramethyl-1′-([1-(2-methoxyethyl)-2-methylimidazolium-3-yl]methyl)-3′-(3-[1-(2-methoxyethyl)-2-methylimidazolium-3-yl]propyl)disiloxane bis(trifluoromethylsulfonyl)imide* (**12**). The yield of IL 12 was 92%. Light yellow liquid. ^1^H NMR (DMSO-d_6_, 300.13 MHz) δ 0.08 (s, 6H), 0.15 (s, 6H), 0.47 (m, 2H), 1.69 (m, 2H), 2.56 (s, 3H), 2.61 (s, 3H), 3.24 (s, 3H), 3.25 (s, 3H), 3.64 (m, 4H), 3.82 (s, 2H), 4.09 (t, J = 7.2 Hz, 2H), 4.32 (t, J = 4.9 Hz, 4H), 7.45 (m, 1H), 7.65 (m, 1H + 2H). ^13^C NMR (DMSO-d_6_, 75.47 MHz) δ −1.08, −0.52, 9.24, 9.45, 13.83, 23.28, 40.09, 47.56, 47.69, 50.11, 58.09, 58.11, 69.88, 70.07, 113.16 * (q *, J_F_ = 321.6 Hz), 117.43 *, 121.06 *, 121.22, 121.47, 121.62, 121.72, 125.95*, 143.58, 144.27. IRS (ATR) 3183, 3146, 3085, 2956, 2940, 2902, 2840, 2822, 1566, 1531, 1453, 1348, 1330, 1260, 1225, 1177, 1134, 1101, 1051, 1014, 919, 838, 803, 789, 762, 739, 672, 653, 613, 599, 569, 510, 466, 406. Gross formula: C_26_H_44_N_6_O_11_F_12_S_4_Si_2_, molecular weight 1029.08. Calculated, %: C 30.35, H 4.31, N 8.17, F 22.15, S 12.46, Si 5.46. Found, %: C 30.22, H 4.47, N 8.11, F 22.13, S 12.38, Si 5.53.

### 3.4. Synthesis of ILs with 2-Hydroxyethyl Group (ILs 7, 8, and 9)

#### General Method

The first stage is the quaternization of 1-(2-hydroxyethyl)imidazole.

1-(2-Hydroxyethyl)imidazole was quaternized with symmetrical and asymmetric 1,1,3,3-tetramethyl-1,3-(3-chloroalkyl)disiloxanes in acetonitrile (50% solution) at an equimolar ratio of the initial reagents for 72 h at the boiling point of the solvent. Then the solvent was removed by rotary evaporation, and the residue (viscous liquid) was dried in a vacuum.

According to NMR spectroscopy (Figure 4), an attempt to prepare chloride precursors of ILs 7 and 9 by this method was unsuccessful, as the products decompose during the quaternization reaction.

The second stage is the ion exchange reaction.

An equimolar amount of lithium bis(trifluoromethylsulfonyl)imide was added to a 30% solution of chloride precursor of IL 8 in acetonitrile. The solution was stirred for 3 h on a magnetic stirrer at room temperature. After removing lithium chloride and distilling off acetonitrile in a vacuum at room temperature, the crude IL 8 was purified by with water until negative reaction of the wash water to Cl^-^ with AgNO_3_, and then dried in a vacuum (0.01 Torr) at 100 °C for 10 h.

*1′,1′,3′,3′-Tetramethyl-1′,3′-bis[3-(1-(2-hydroxyethyl)imidazolium-3-yl)propyl]disiloxane bis(trifluoromethylsulfonyl)imide* (**8**). The yield of IL 8 was 71%. Colorless liquid. ^1^H NMR (DMSO-d_6_, 300.13 MHz) δ 0.06 (s, 12H), 0.45 (m, 4H), 1.78 (m, 4H), 3.75 (m, 4H), 4.14 (t, J = 7.22 Hz, 4H), 4.22 (t, J = 5.0 Hz, 4H), 5.16 (t, J = 5.2 Hz, 2H), 7.75 (m. 4H), 9.12 (s, 2H). ^13^C NMR (DMSO-d_6_, 75.47 MHz) δ 0.07, 14.9, 23.84, 51.40, 51.69, 59.25, 113.10 * (q *, J_F_ = 328.2 Hz), 117.36 *, 121.62 *, 122.07, 122.79, 125.89 *, 136.30. ^29^Si NMR (59.60 MHz, DMSO-d_6_) δ 8.17. IRS (ATR), cm^−1^: 3537, 3152, 3116, 3095, 2958, 2890, 2809, 1563, 1448, 1407, 1347, 1329, 1256, 1226, 1180, 1133, 1050, 941, 916, 839, 789, 763, 740, 710, 652, 613, 599, 569, 510, 406. Gross formula: C_24_H_40_N_6_F_12_S_4_Si_2_O_11_, molecular weight 1001.03. Calculated (%): C, 28.80; H, 4.03; N, 8.39; F, 22.78; S, 12.81; Si, 5.61. Found (%): C, 28.71; H, 4.11; N, 8.32; F, 22.75; S, 12.77; Si, 5.69.

## 4. Conclusions

The present study of the properties of a number of bis(trifluoromethylsulfonyl)imide disiloxane-based dicationic ILs with different linker structures and the nature of substituents in the cation shows that the nature of the substituents in the imidazolium cation significantly affected the properties of ILs. Introduction of polar substituents in the imidazolium cation led to an increase in viscosity and a decrease in the thermal stability of these ILs. The observed increase in volatility of the dicationic ILs with polar groups is explained by the increased rate of thermal decomposition processes, although it is known that OH groups in monocationic imidazolium ionic liquids effectively reduce their volatility [17]. Using the example of 1,2-dimethylimidazolium-based ILs, it can be noted that the length and symmetry of the linker have only a weak effect on the melting point of such ILs. Replacing the methylene spacer with a propylene one in the linker led to a noticeable decrease in the thermal stability of ILs due to an introduction of an aliphatic chain, but helped to increase the hydrolytic stability of the siloxane bond by decreasing its polarity. The presence of methyl groups at the position 2 of the imidazolium cation had a significant effect on the viscosity and melting points of the resulting ILs. The dicationic ILs prepared from 1-methylimidazole had lower viscosity (300 cSt at 30 °C) and low glass transition temperatures (<−50 °C); the decomposition temperature (according to TGA) exceeded 400°C. Their volatility in a vacuum was <1 mg·h^−1^·cm^−2^.

## Figures and Tables

**Table 1:** The properties of disiloxane dicationic ionic liquids 1–12 with bis(trifluoromethylsulfonyl)imide anion.

IL	Cation Structure (Scheme 1)	T_d_, °C (TGA)	T_m_, °C (DSC)	Volatility, mg·h^−1^·cm^−2^ at 150 °C/190 °C/220 °C **
1	*n* = 1, m = 1, R_1_ = R_2_ = CH_3_	437	69	0.00/0.03/0.04
2	*n* = 3, m = 3, R_1_ = R_2_ = CH_3_	419	59	0.01/0.02/0.17
3	*n* = 1, m = 3, R_1_ = R_2_ = CH_3_	420	42	0.02/0.11/0.40
4	*n* = 1, m = 1, R_1_ = CH_3_, R_2_ = H	435	−52 *	0.02/0.08/0.22
5	*n* = 3, m = 3, R_1_ = CH_3_, R_2_ = H	402	−58 *	0.03/0.33/0.95
6	*n* = 1, m = 3, R_1_ = CH_3_, R_2_ = H	410	−56 *	0.08/0.59/2.44
7	*n* = 1, m = 1, R_1_ = CH_2_CH_2_OH, R_2_ = H	***		
8	*n* = 3, m = 3, R_1_ = CH_2_CH_2_OH, R_2_ = H	391	−53 *	0.02/0.12/0.35
9	*n* = 1, m = 3, R_1_ = CH_2_CH_2_OH, R_2_ = H	***		
10	*n* = 1, m = 1, R_1_ = CH_2_CH_2_OCH_3_, R_2_ = CH_3_	438	−43 *	0.07/0.14/0.29
11	*n* = 3, m = 3, R_1_ = CH_2_CH_2_OCH_3_, R_2_ = CH_3_	416	−48 *	0.02/0.22/0.94
12	*n* = 1, m = 3, R_1_ = CH_2_CH_2_OCH_3_, R_2_ = CH_3_	421	−47 *	0.03/0.31/0.76

* T_g_—glass transition temperature; ** in vacuum 1 × 10^−4^ Torr; *** the synthesis via the proposed scheme was impossible.

**Table 2:** Viscosity of dicationic disiloxane ILs 4–6, 8, and 10–12 and parameters of the Vogel–Tamman–Fulcher equation (R^2^ > 0.99).

IL	Viscosity at 30 °C, cSt	Parameters of the Vogel–Tamman–Fulcher equation *		
		a	-b	T_0_
4	312	798.0	1.402	191.5
5	344	901.3	1.681	183.3
6	242	797.4	1.408	187.5
8	602	863.0	1.451	193.4
10	683	765.7	1.372	206.0
11	555	890.9	1.784	193.3
12	563	792.7	1.491	202.0

* Vogel–Tamman–Fulcher equation: lnν = b + [a/(T − T_0_)].

## Supplementary Materials

The following are available online. General method for the synthesis of symmetric disiloxane dicationic ILs 1, 2, 4, 5, 10 and 11; Synthesis of 1-(2-methoxyethyl)-2-methylimidazole; Experimental data of ILs with an asymmetric linker.
