# Supplementary material for: Properties of Dicationic Disiloxane Ionic Liquids
Source: Molecules. 2020 Jun 26;25(12):2949. doi: 10.3390/molecules25122949 (PMC7355833; doi:10.3390/molecules25122949)
Supplement: Supplementary file 1 [file molecules-25-02949-s001.pdf]

Supplementary Material

Properties of Dicationic Disiloxane Ionic Liquids

Vladimir G. Krasovskiy [1,*], Gennady I. Kapustin [1], Olga B. Gorbatsevich [2], Lev M. Glukhov [1], Elena A. Chernikova [1], Anatoly A. Koroteev [3] and Leonid M. Kustov [1,4,5,*]

[1] N. D. Zelinsky Institute of Organic Chemistry, Russian Academy of Sciences, Russian Federation, Leninsky prosp. 47, 119991 Moscow, Russia; gik@ioc.ac.ru (G.I.K.); elektron77@mail.ru (L.M.G.); chernikova_e@mail.ru (E.A.C.)

[2] N. S. Enikolopov Institute of Synthetic Polymer Materials, Russian Academy of Sciences, Russian Federation, ul. Profsoyuznaya 70, 117393 Moscow, Russia; olborg@list.ru

[3] Moscow Aviation Institute, Russian Federation, Volokolamskoe Shosse 4, 125993 Moscow, Russia; chkt@yandex.ru

[4] Chemistry Department, Moscow State University, Russian Federation, Leninskie Gory 1, bldg 1., 119992 Moscow, Russia

[5] National University of Science and Technology MISiS, Moscow, Leninsky prosp. 4, 119991 Moscow, Russia

* Correspondence: miyusha@mail.ru (V.G.K); lmkustov@mail.ru (L.M.K)

Table of contents

1. General method for the synthesis of symmetrical disiloxane dicationic ILs 1, 2, 4, 5, 10 and 11 — S2

2. Synthesis of 1-(2-methyoxyethyl)-2-methylimidazole — S2

3. Experimental data of ILs with asymmetric linkers (3, 6, and 12) — S3

3.1. 1’,1’,3’,3’-Tetramethyl-1’-([1,2-dimethylimidazolium-3-yl]methyl)-3’-(3-[1,2-dimethylimidazolium-3-yl]propyl)disiloxane bis(trifluoromethylsulfonyl)imide (3). — **S3**

3.2. 1’,1’,3’,3’-Tetramethyl-1’-([1-methylimidazolium-3-yl]methyl)-3’-(3-[1-methylimidazolium-3-yl]propyl)disiloxane bis(trifluoromethylsulfonyl)imide (6). — **S7**

3.3. 1’,1’,3’,3’-Tetramethyl-1’-([1-(2-methoxyethyl)-2-methylimidazolium-3-yl]methyl)-3’-(3-[1-(2-methoxyethyl)-2-methylimidazolium-3-yl]propyl)disiloxane bis(trifluoromethylsulfonyl)imide (12). — **S10**

4. Experimental data of OH-containing IL — S13

1’,1’,3’,3’-Tetramethyl-1’,3’-bis[3-(1-(2-hydroxyethyl)imidazolium-3-yl)propyl] disiloxane (8) — **S13**

1. General method for the synthesis of symmetric disiloxane dicationic ILs 1, 2, 4, 5, 10 and 11 [18,19].

The first stage is the synthesis of symmetric linkers

The symmetric linkers – 1,1,3,3-tetramethyl-1,3-di(chloromethyl)disiloxane and 1,1,3,3-tetramethyl-1,3-di(3-chloropropyl)disiloxane – were synthesized by hydrolytic condensation of dimethyl(chloromethyl)chlorosilane or dimethyl(3-chloropropyl)chlorosilane with water at the reagents volume ratio 1:3, respectively. Chlorosilane was added to water and, after 1 hour stirring at room temperature, hexane was added in a volume equal to the volume of water. The organic layer was separated, washed with water to a neutral medium and dried from traces of water with sodium sulfate for 2 days. Hexane was removed. The yield of the target products was 96-98%. The purity of the products according to GC was >97%.

The second stage is the synthesis of chloride ILs 1, 2, 4, 5, 10, and 11.

An alkyl-substituted imidazole (1,2-dimethylimidazole, 1-methylimidazole, and 1-(2-methoxyethyl)-2-methylimidazole) was quaternized with a symmetrical disiloxane in acetonitrile (50% solution) at an equimolar ratio of the initial reagents at boiling temperature for 72 hours. After that, the precipitate was collected and dried in a vacuum.

The third stage is the synthesis of bis(trifluoromethylsulfonyl)imide ILs 1, 2, 4, 5, 10, and 11.

The mixture of the chloride precursor obtained at the previous stage and a 30% solution of lithium bis(trifluoromethylsulfonyl)imide (10% excess) in acetonitrile was stirred for 90 minutes. Then acetonitrile was removed, bis(trifluoromethylsulfonyl)imide IL was dissolved in dichloromethane (30% solution) and the solution was extracted with small amounts of water until the negative reaction of the wash water with $AgNO_3$. The ionic liquid was dried by azeotropic distillation of dry dichloromethane (100 ml CH_2Cl_2 to 1 g of IL).

The experimental data for ILs 1, 2, 4, 5, 10, and 11 are presented in the Supplementary Material for articles [18,19].

2. Synthesis of 1-(2-methoxyethyl)-2-methylimidazole [19].

1-(2-methoxyethyl)-2-methylimidazole was synthesized in two stages by alkylation of 2-methylimidazole with 1-chloro-2-methoxyethane (scheme S1). First, from equimolar amounts of 2-methylimidazole and potassium tert-butylate in acetonitrile (50% solution) at room temperature for 1 hour, the corresponding potassium imidazolate was obtained. After adding an equimolar amount of 1-chloro-2-methoxyethane, the reaction mixture was stirred for 8 hours at room temperature. The precipitated potassium chloride was filtered and the solvent was removed

in a vacuum. The target product was isolated by rectification with a yield of 58 wt. % (b. p. = 89-90°C/0.1 Torr). The purity of the resulting product according to GC data was 98%.

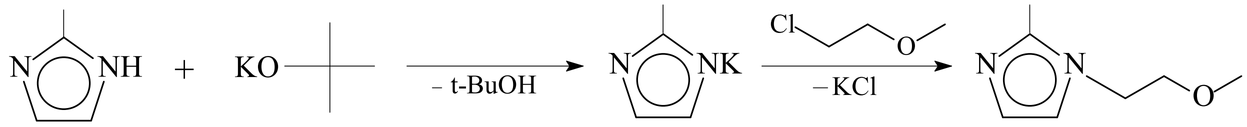

Scheme S1. The synthesis of 1-(2-methoxyethyl)-2-methylimidazole.

3. Experimental data of ILs with an asymmetric linker (3, 6, and 12)

3.1. *1',1',3',3'-Tetramethyl-1'-([1,2-dimethylimidazolium-3-yl]methyl)-3'-(3-[1,2-dimethylimidazolium-3-yl]propyl)disiloxane bis(trifluoromethylsulfonyl)imide (3).*

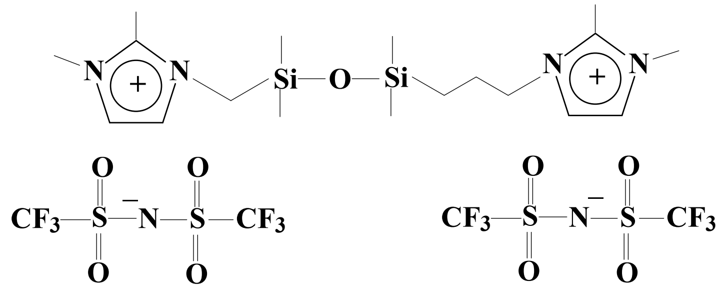

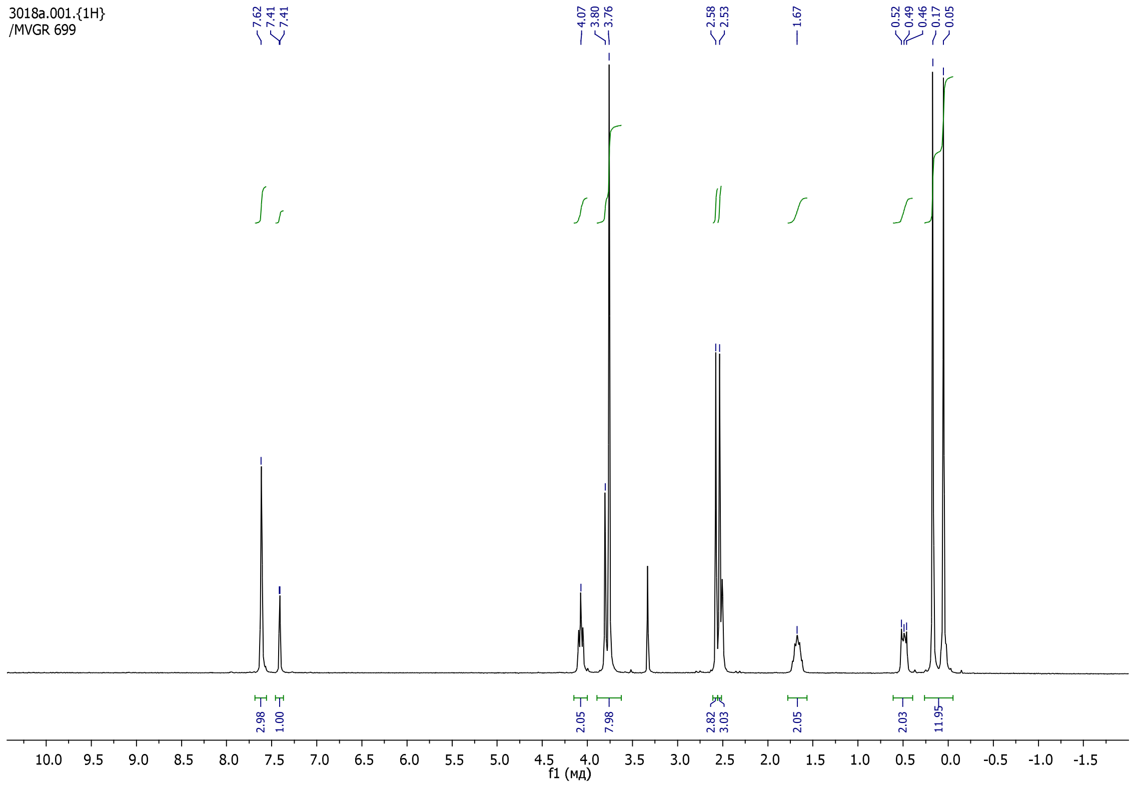

Figure S1. ^{1}H NMR spectrum of IL 3.

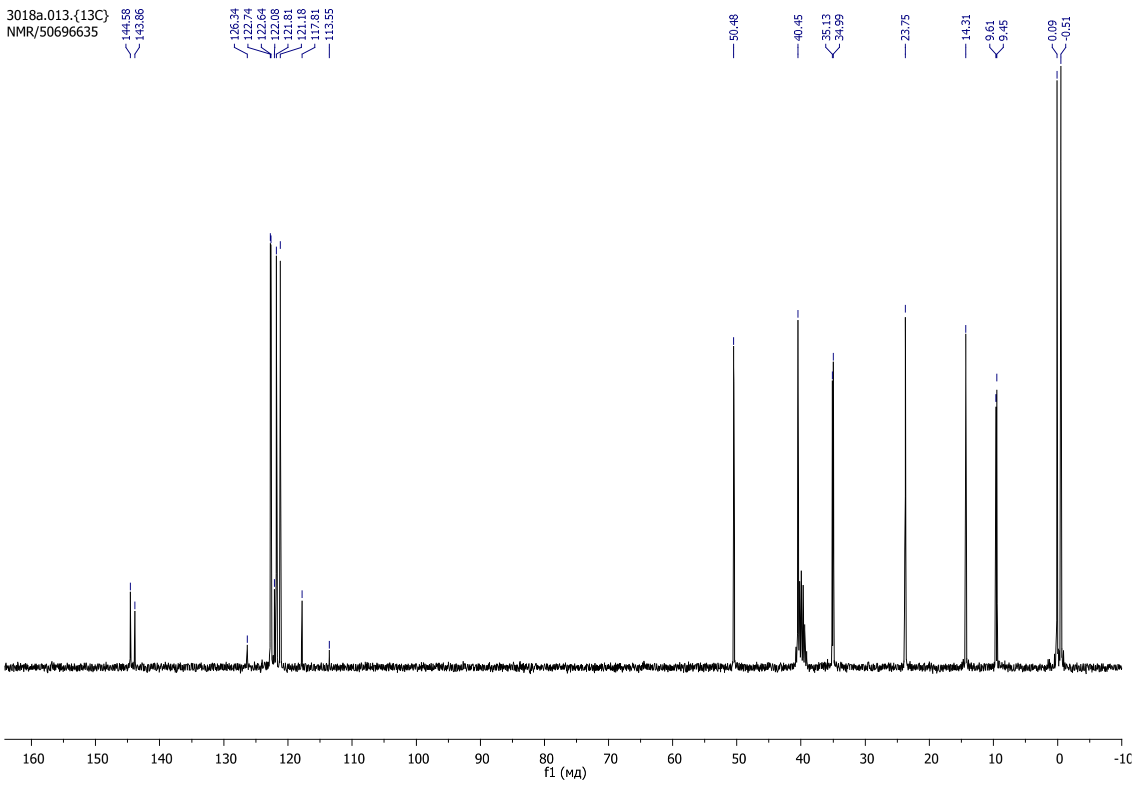

Figure S2. ^{13}C NMR spectrum of IL 3.

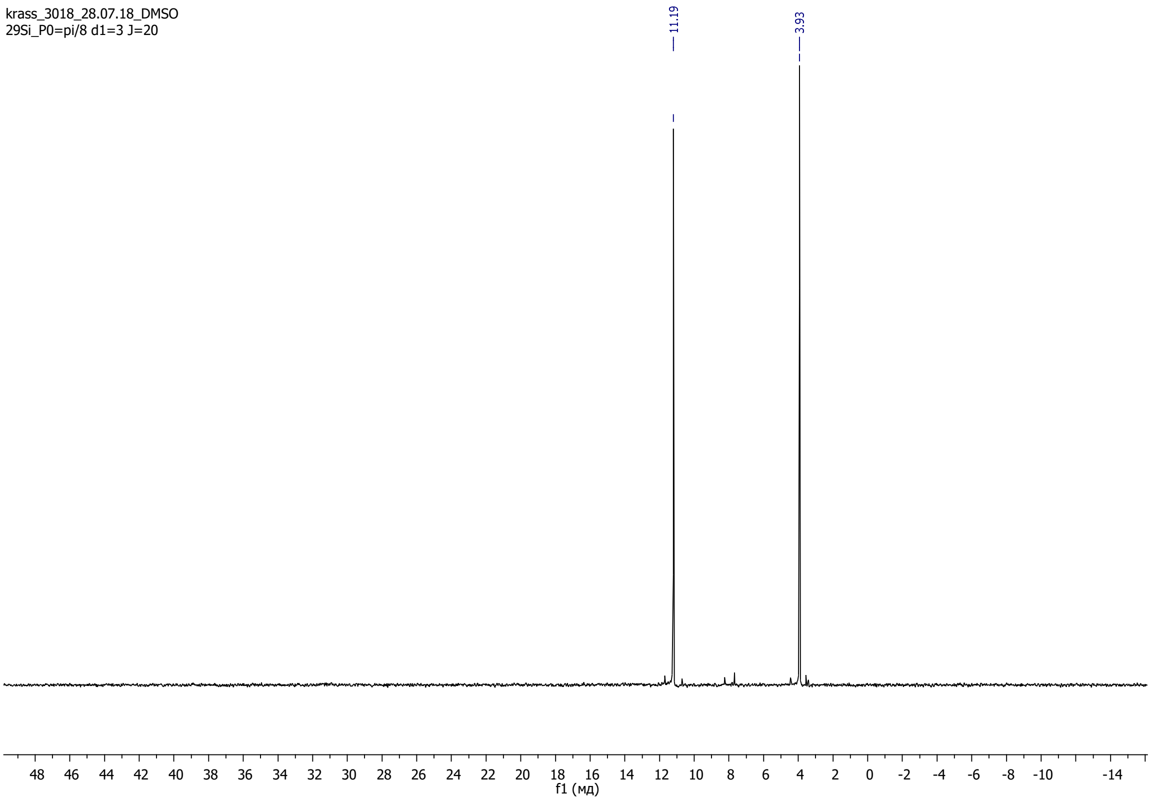

Figure S3. ^{29}Si NMR spectrum of IL 3.

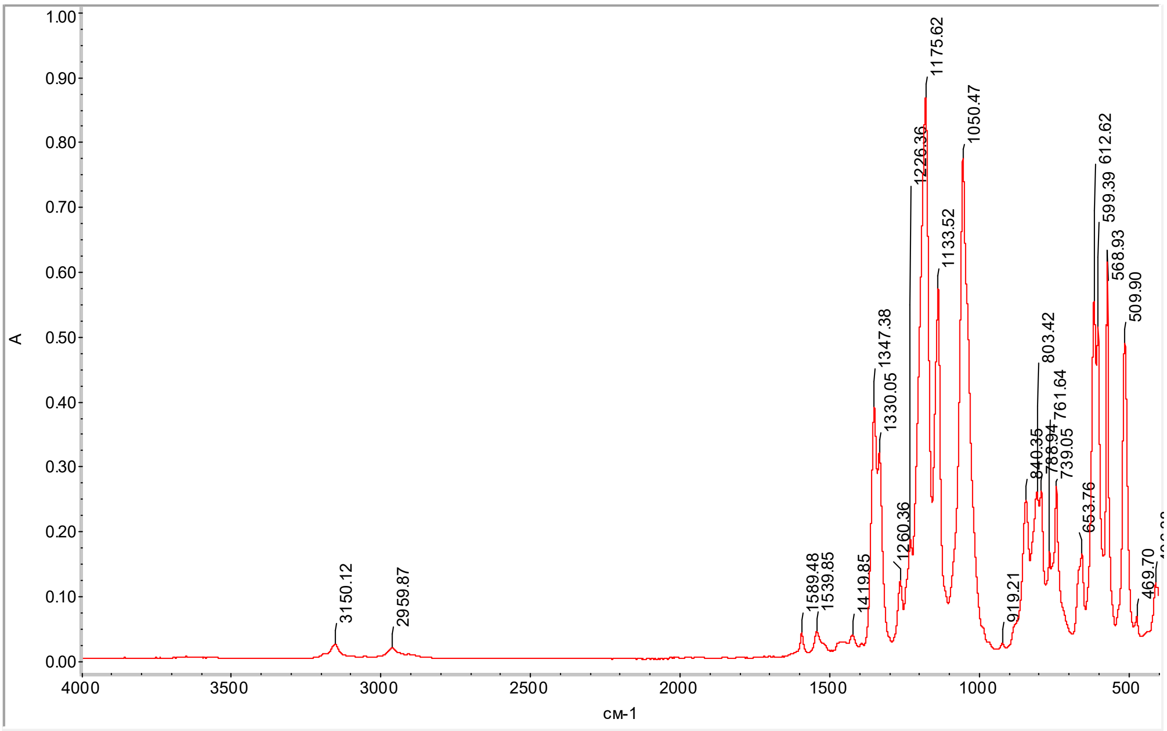

Figure S4. IR spectrum (ATR) of IL 3.

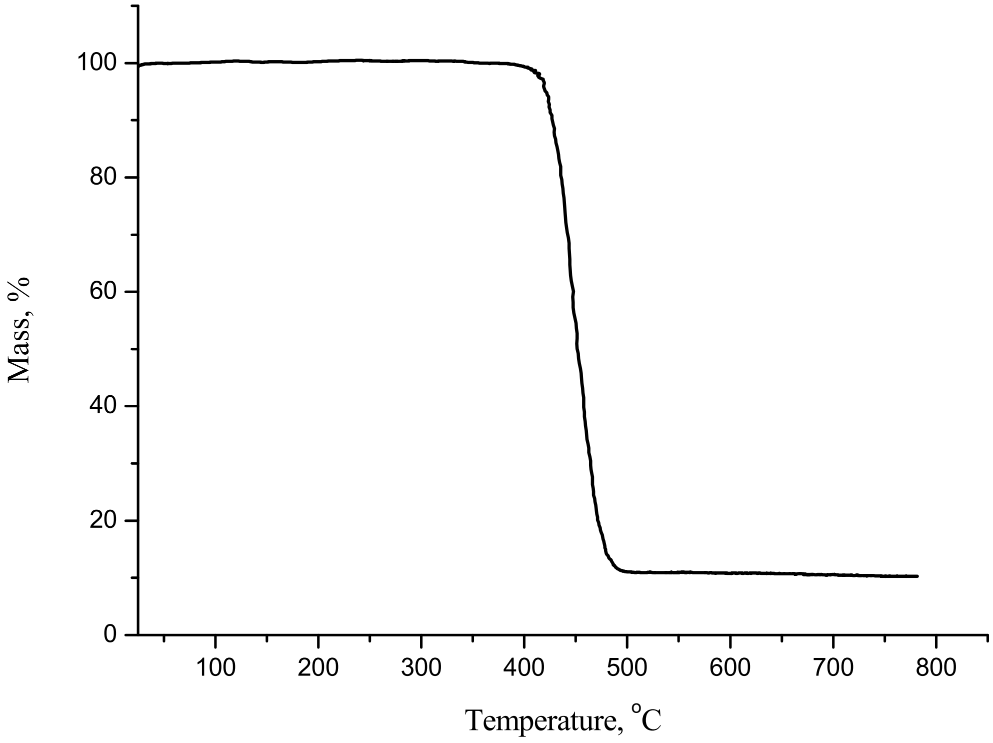

Figure S5. The thermogram of IL 3.

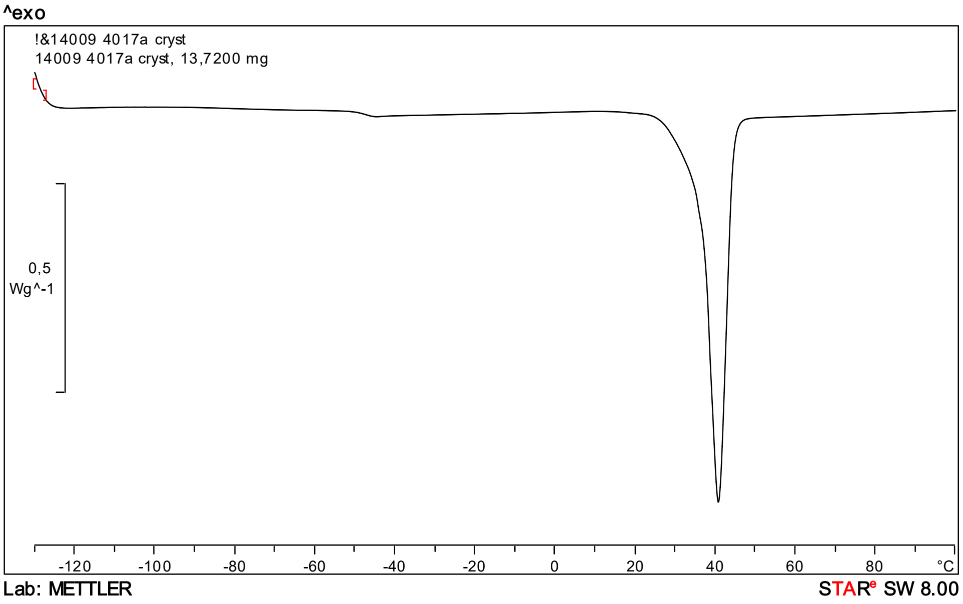

Figure S6. DSC curve of IL 3.

3.2. *1',1',3',3'-Tetramethyl-1'-([1-methylimidazolium-3-yl]methyl)-3'-(3-[1-methylimidazolium-3-yl]propyl)disiloxane bis(trifluoromethylsulfonyl)imide (6).*

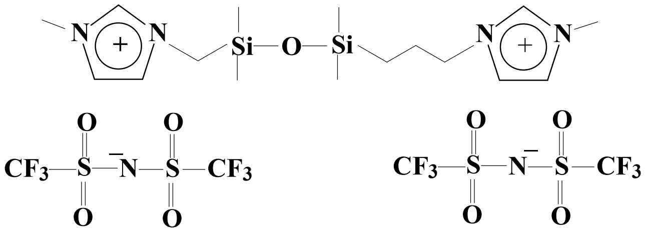

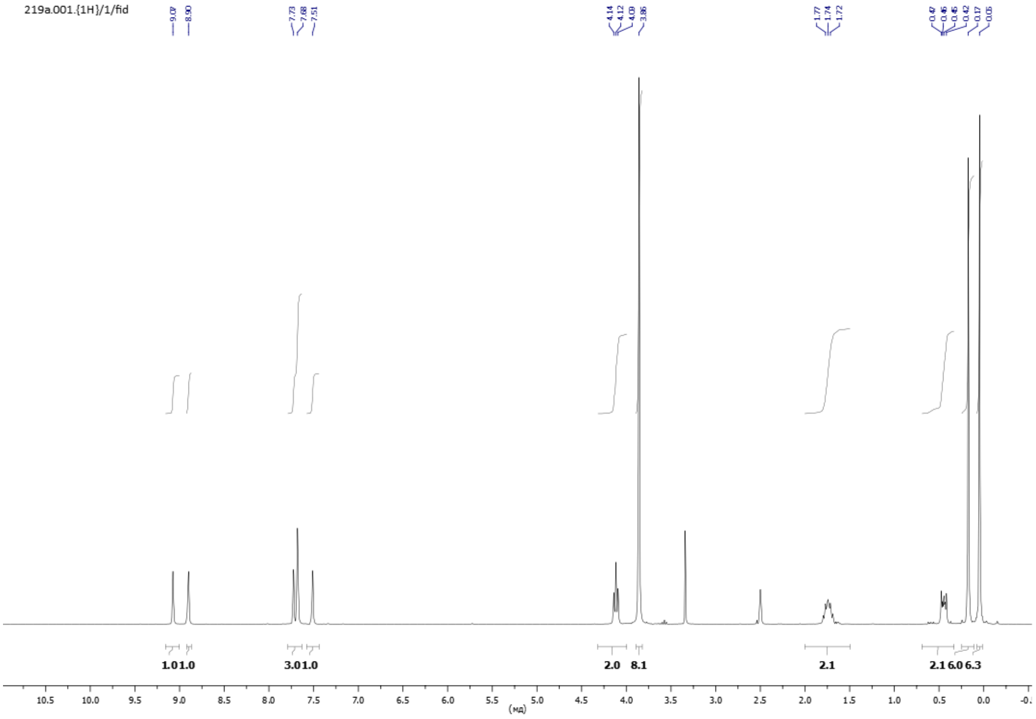

Figure S7. ^{1}H NMR spectrum of IL 6.

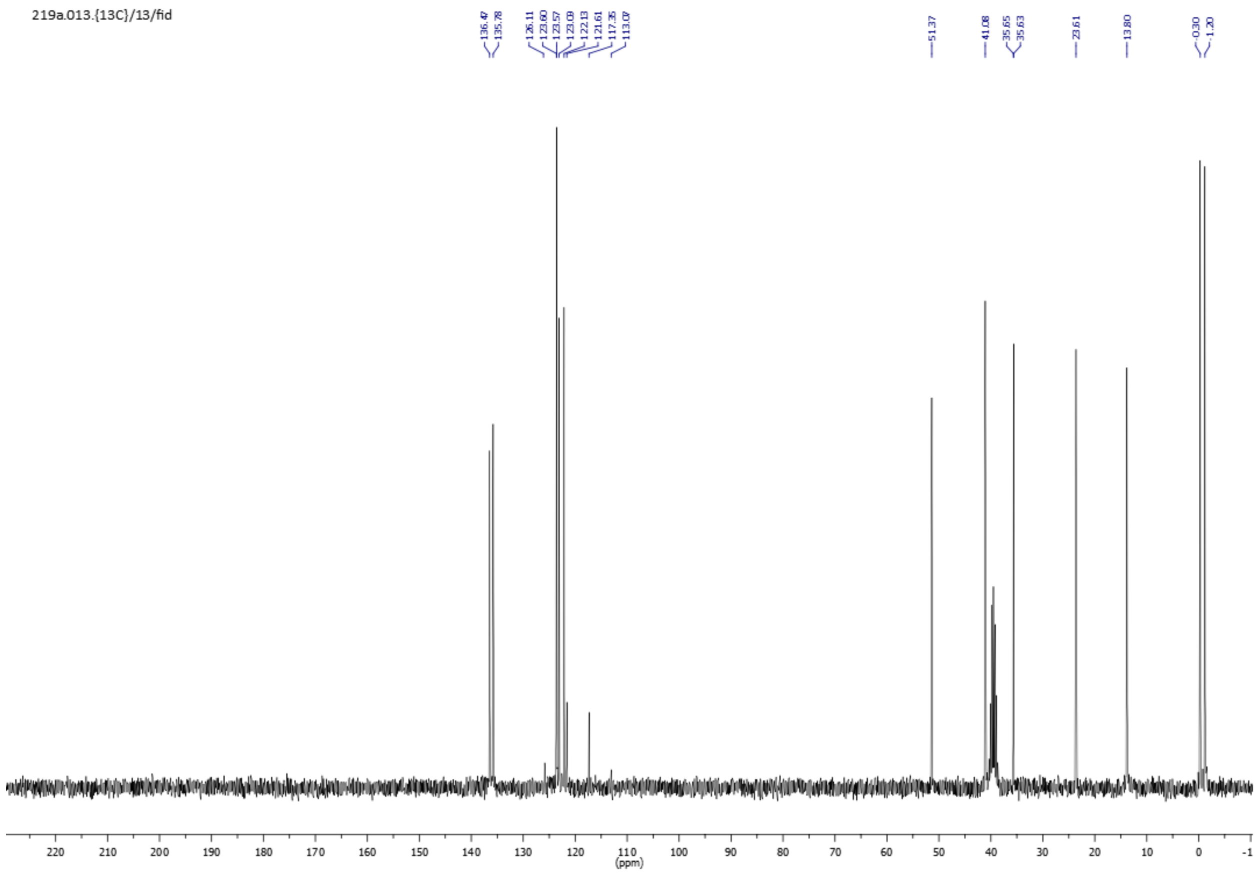

Figure S8. ^{13}C NMR spectrum of IL 6.

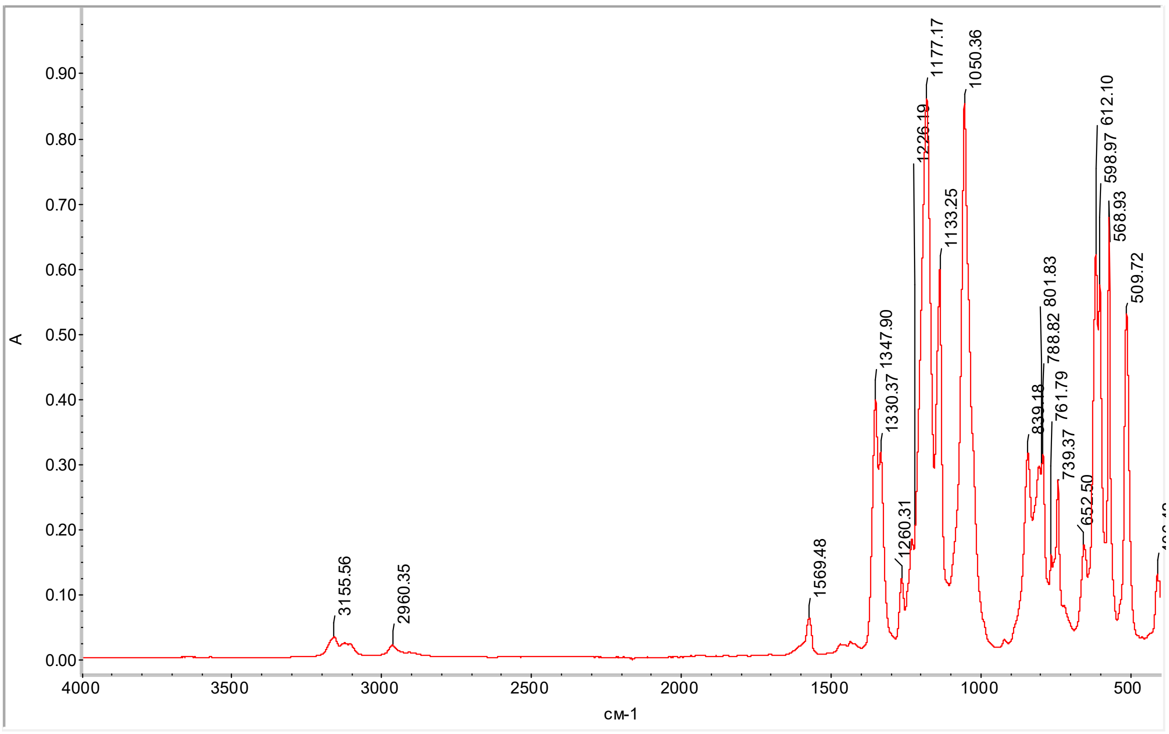

Figure S9. IR spectrum (ATR) of IL 6.

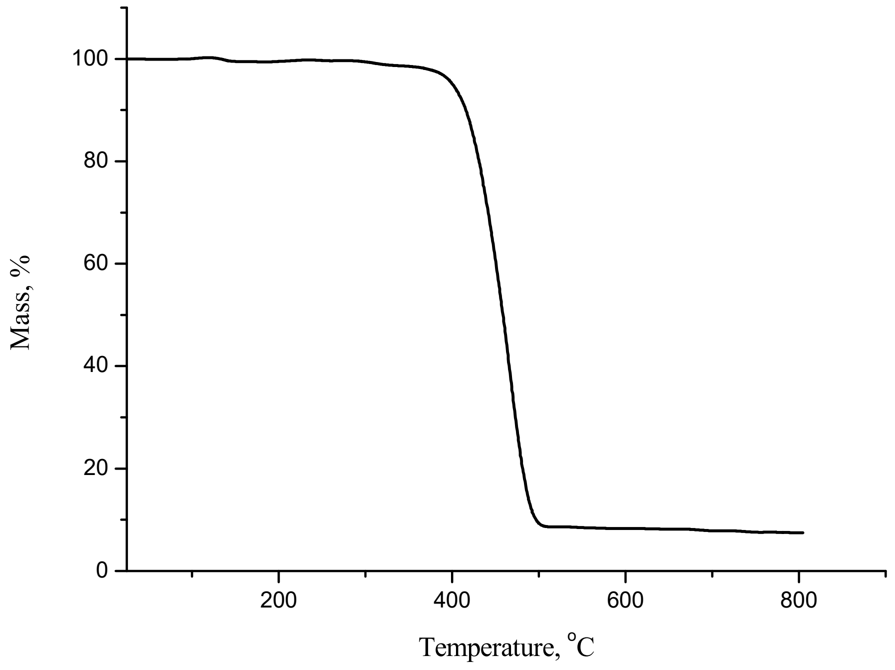

Figure S10. The thermogram of IL 6.

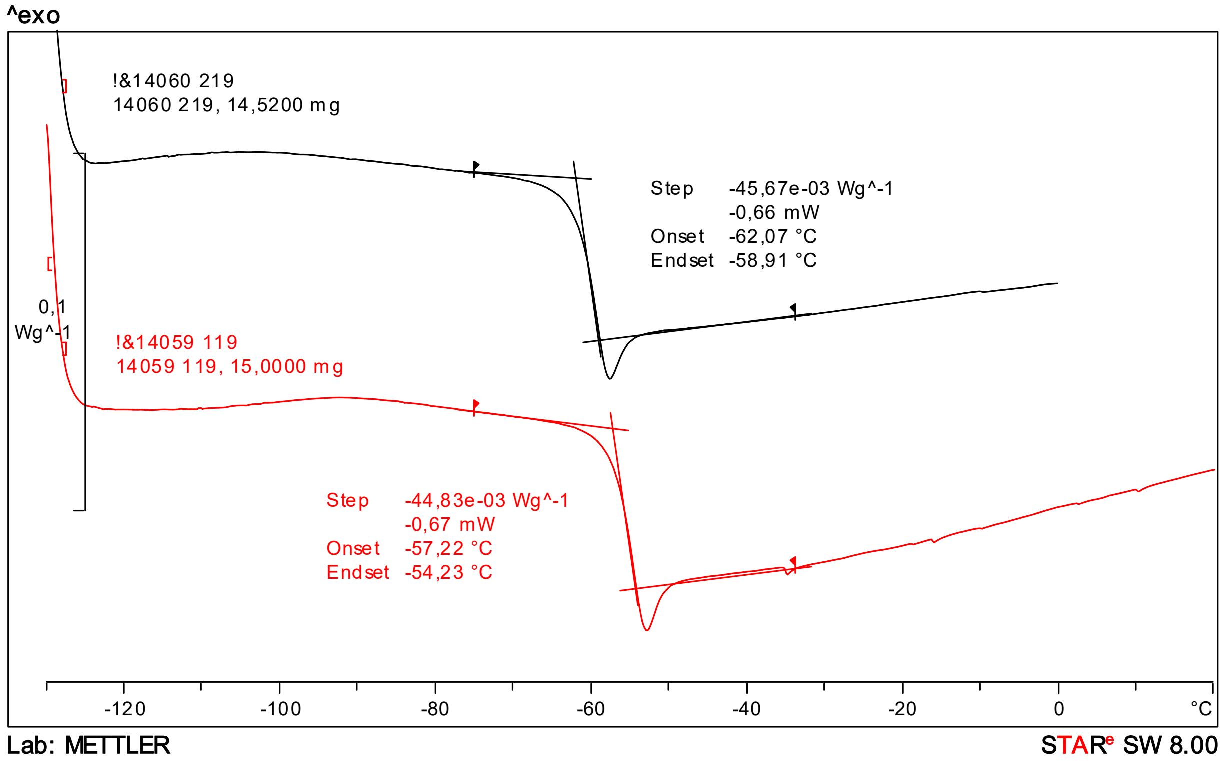

Figure S11. DSC curve of IL 6 (black).

3.3. *1',1',3',3'-Tetramethyl-1'-([1-(2-methoxyethyl)-2-methylimidazolium-3-yl]methyl)-3'-(3-[1-(2-methoxyethyl)-2-methylimidazolium-3-yl]propyl)disiloxane bis(trifluoromethylsulfonyl)imide (12).*

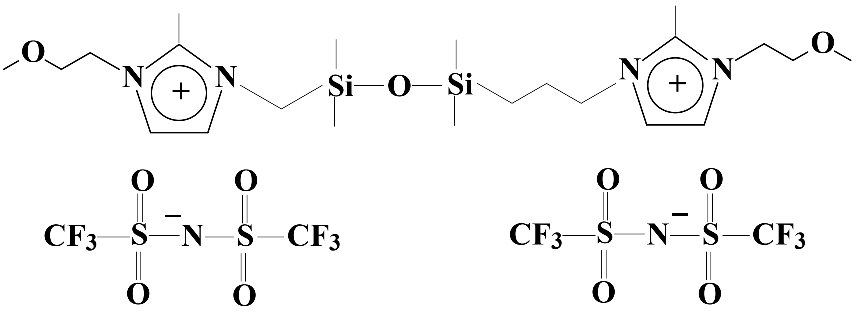

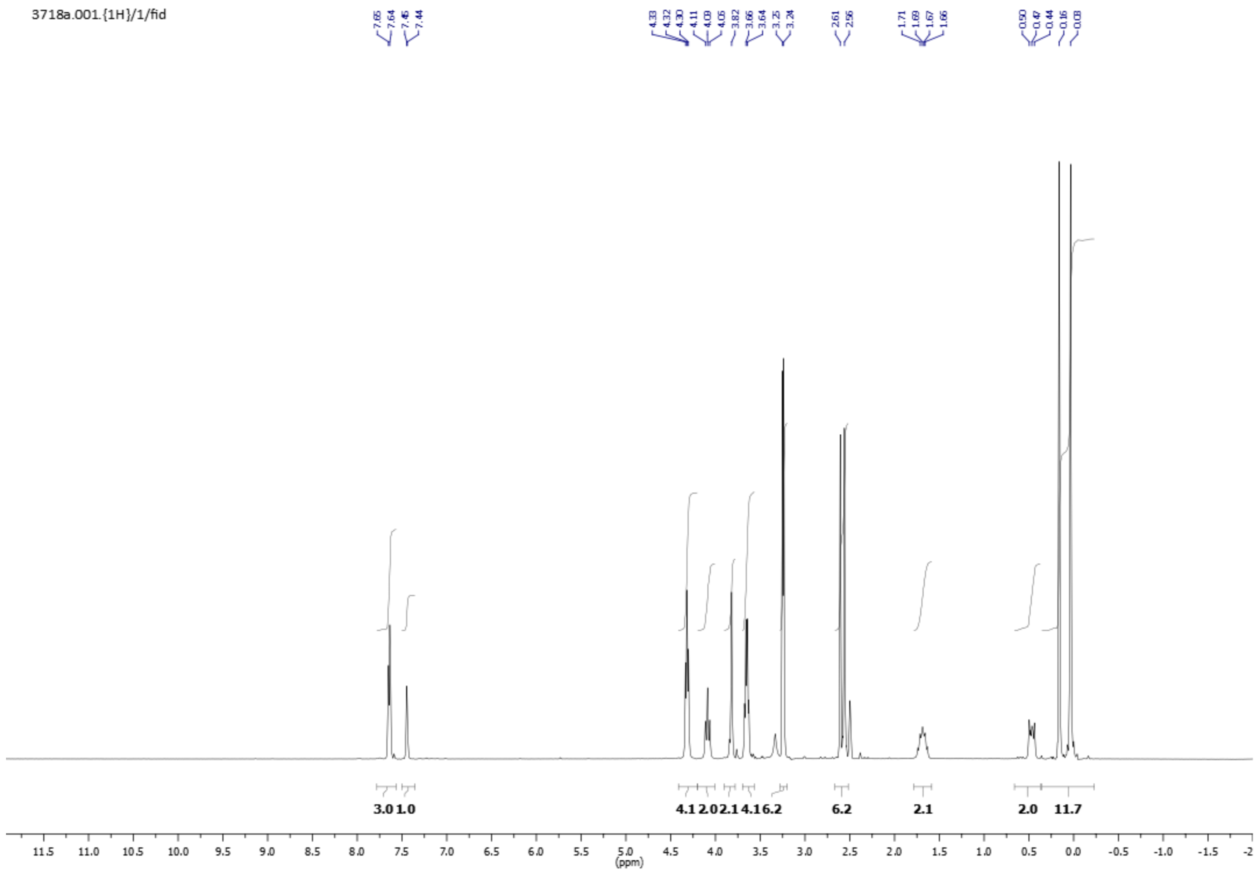

Figure S12. ^{1}H NMR spectrum of IL 12.

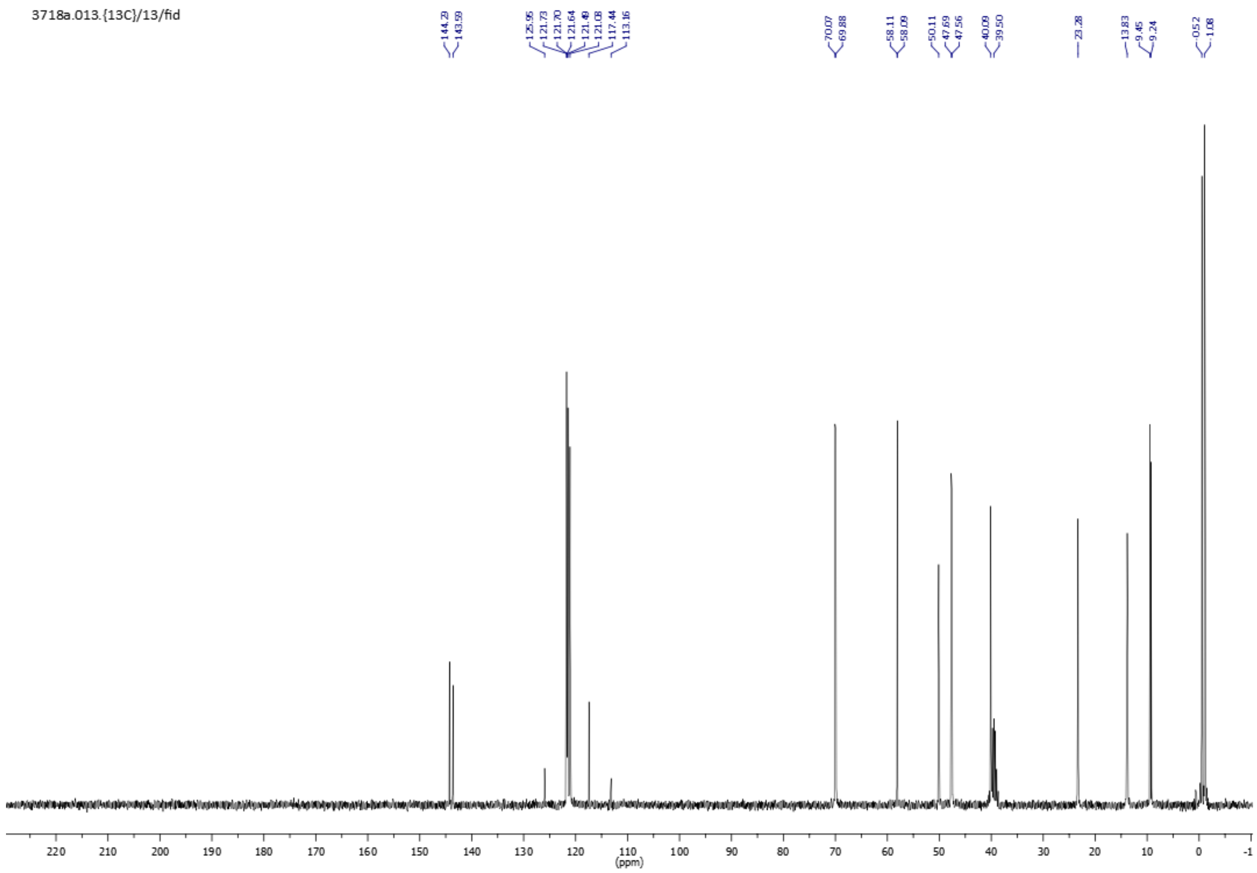

Figure S13. ^{13}C NMR spectrum of IL 12.

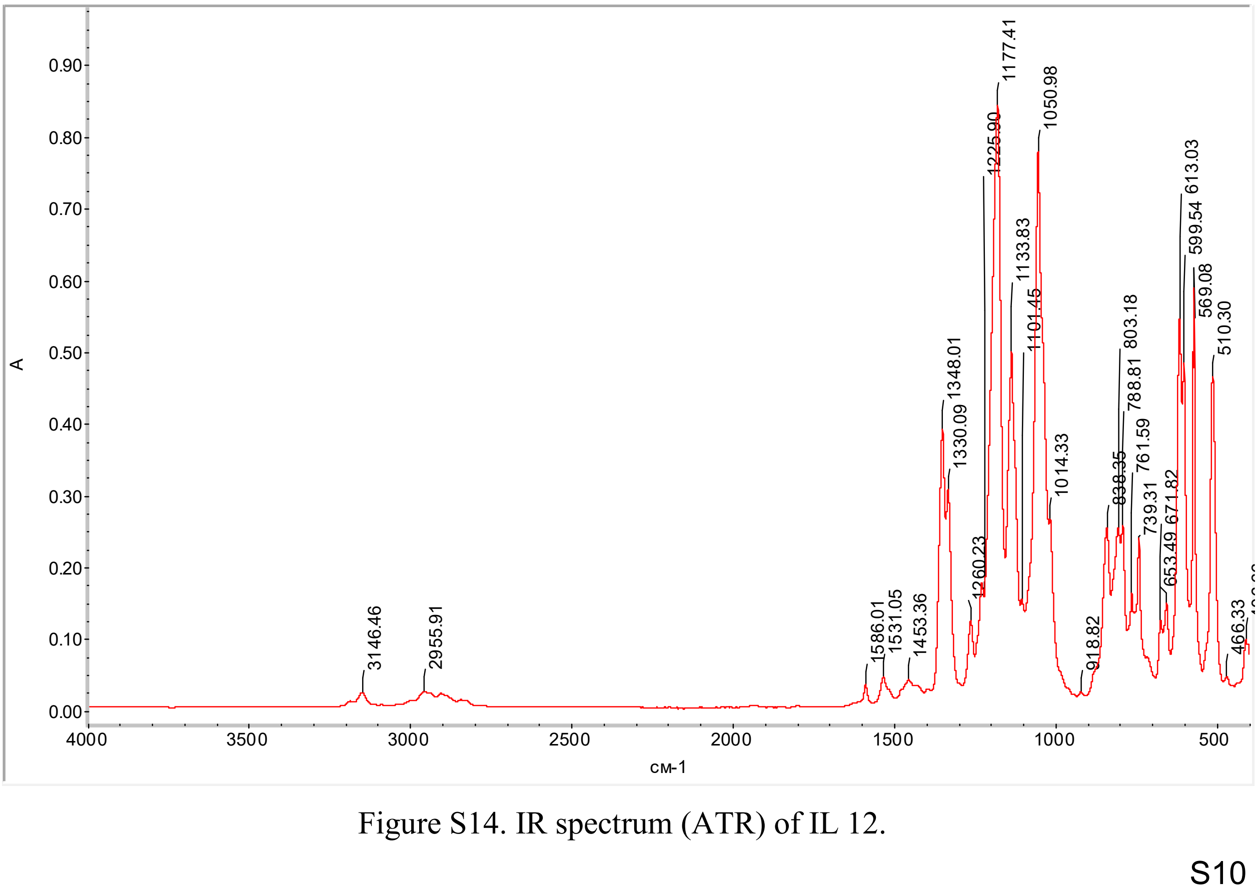

Figure S14. IR spectrum (ATR) of IL 12.

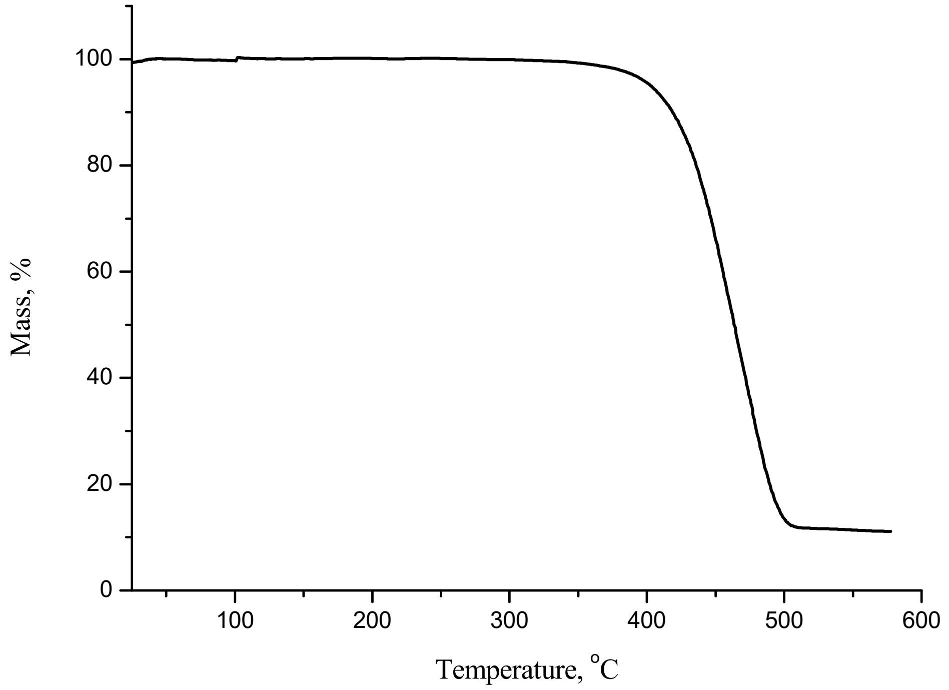

Figure S15. The thermogram of IL 12.

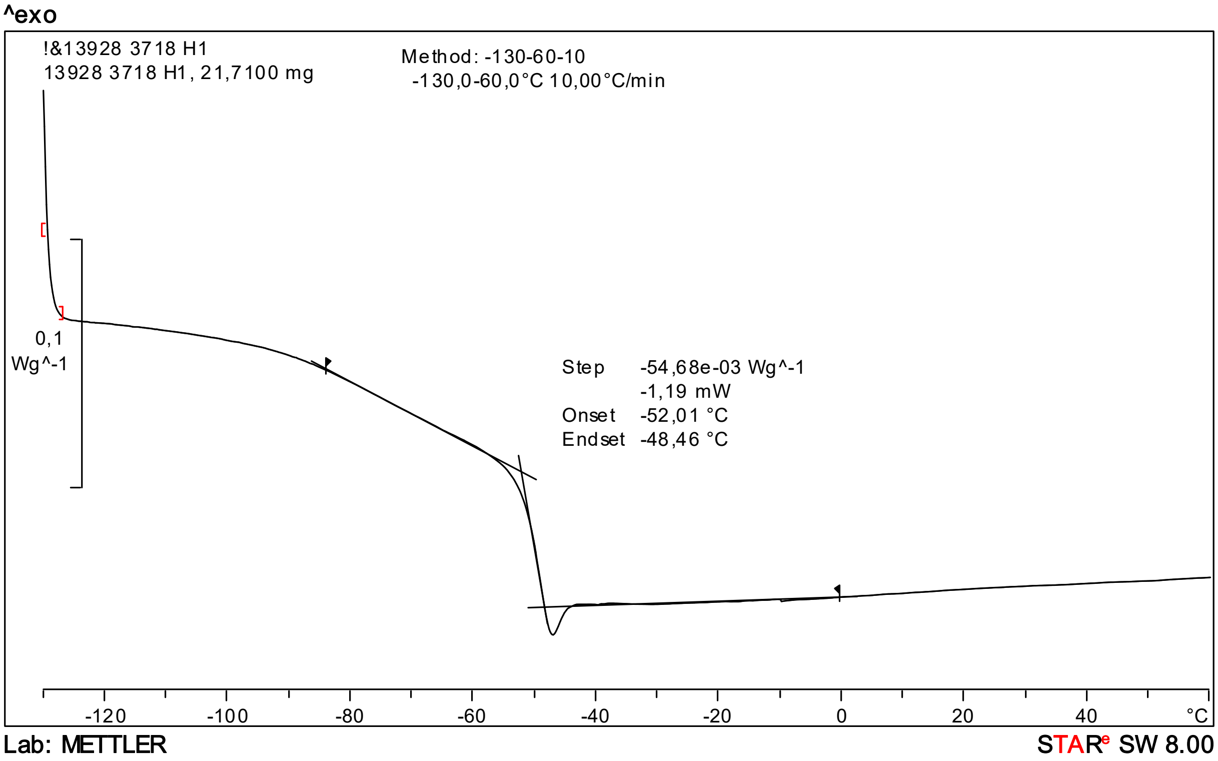

Figure S16. DSC curve of IL 12.

4. Experimental data of IL OH-containing IL 8

1',1',3',3'-Tetramethyl-1',3'-bis[3-(1-(2-hydroxyethyl)imidazolium-3-yl)propyl] disiloxane (8).

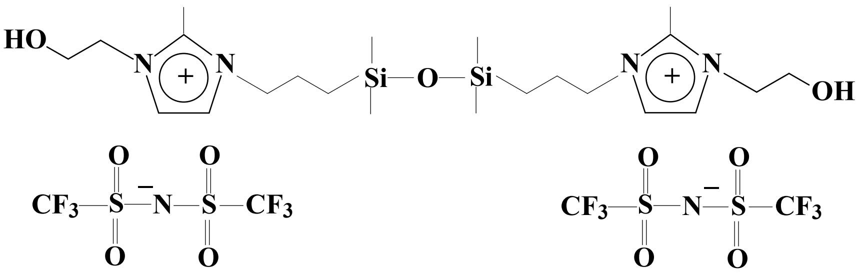

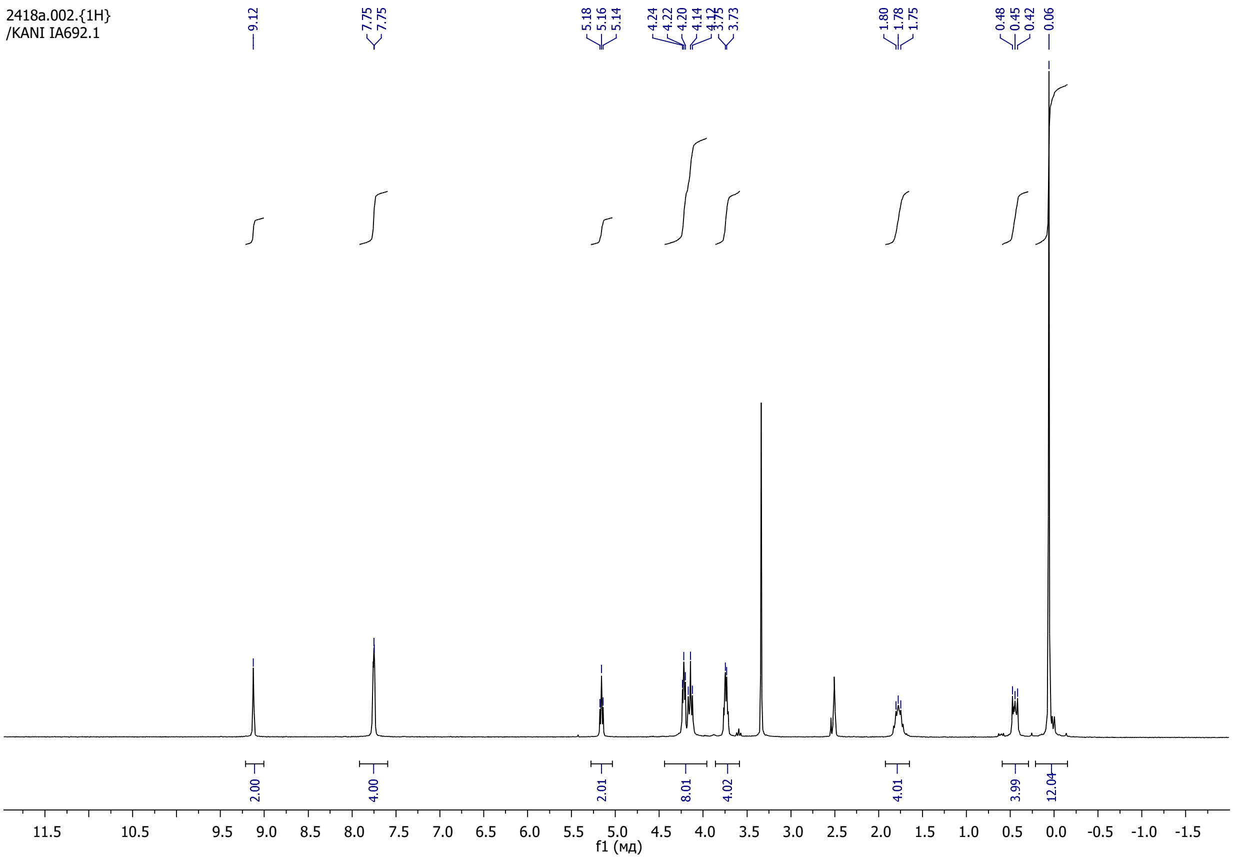

Figure S17. ^{1}H NMR spectrum of IL 8.

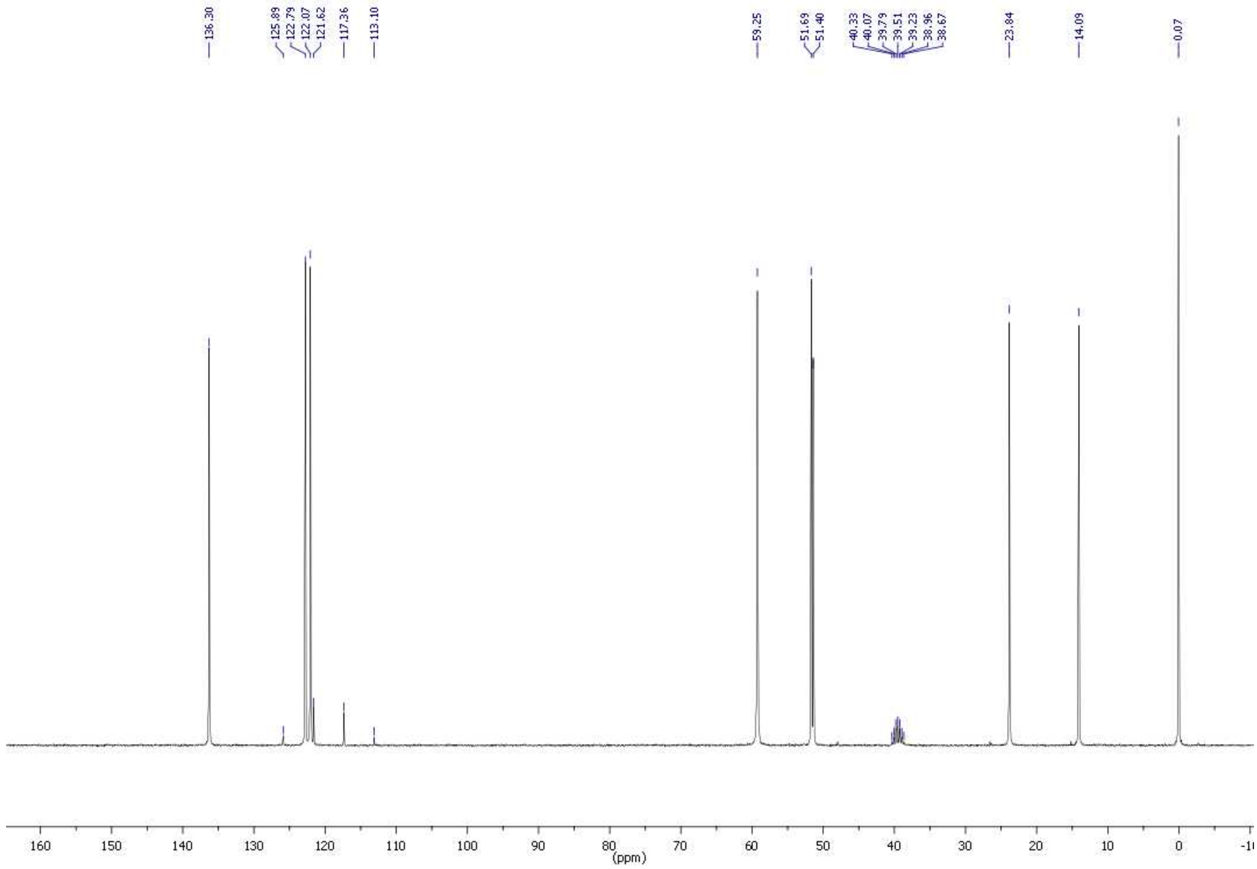

Figure S18. ^{13}C NMR spectrum of IL 8.

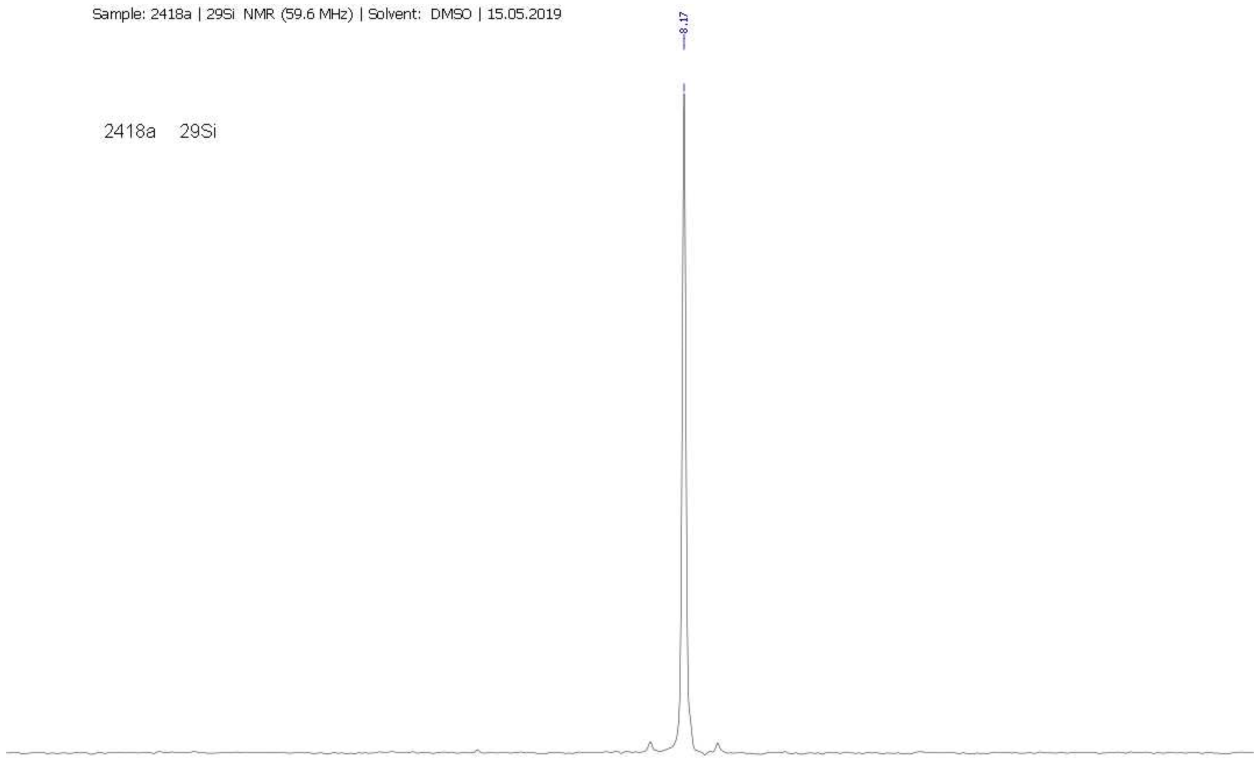

Figure S19. ^{29}Si NMR spectrum of IL 8.

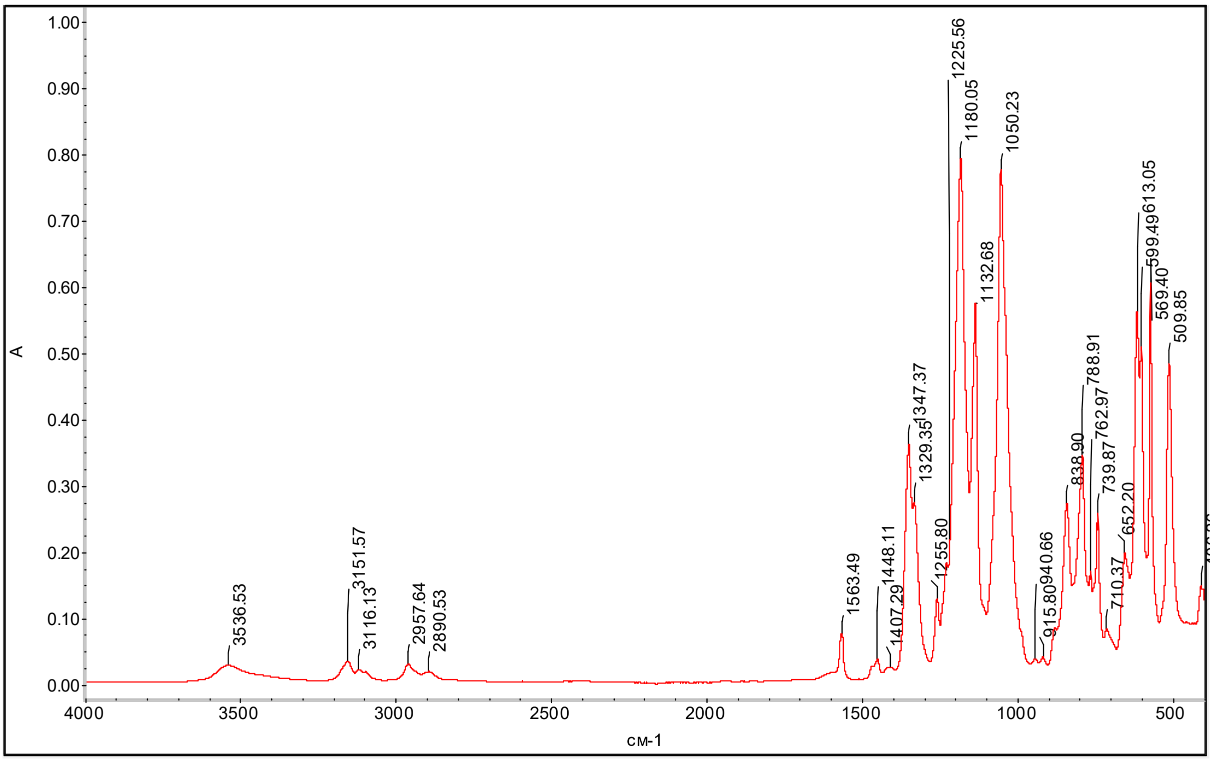

Figure S20. IR spectrum (ATR) of IL 8.

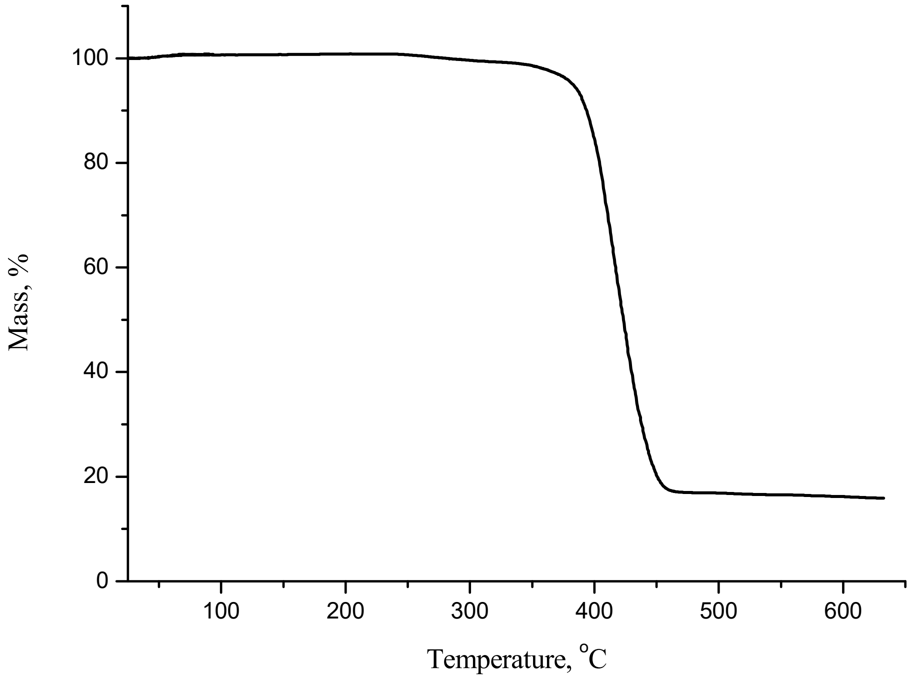

Figure S21. The thermogram of IL 8.

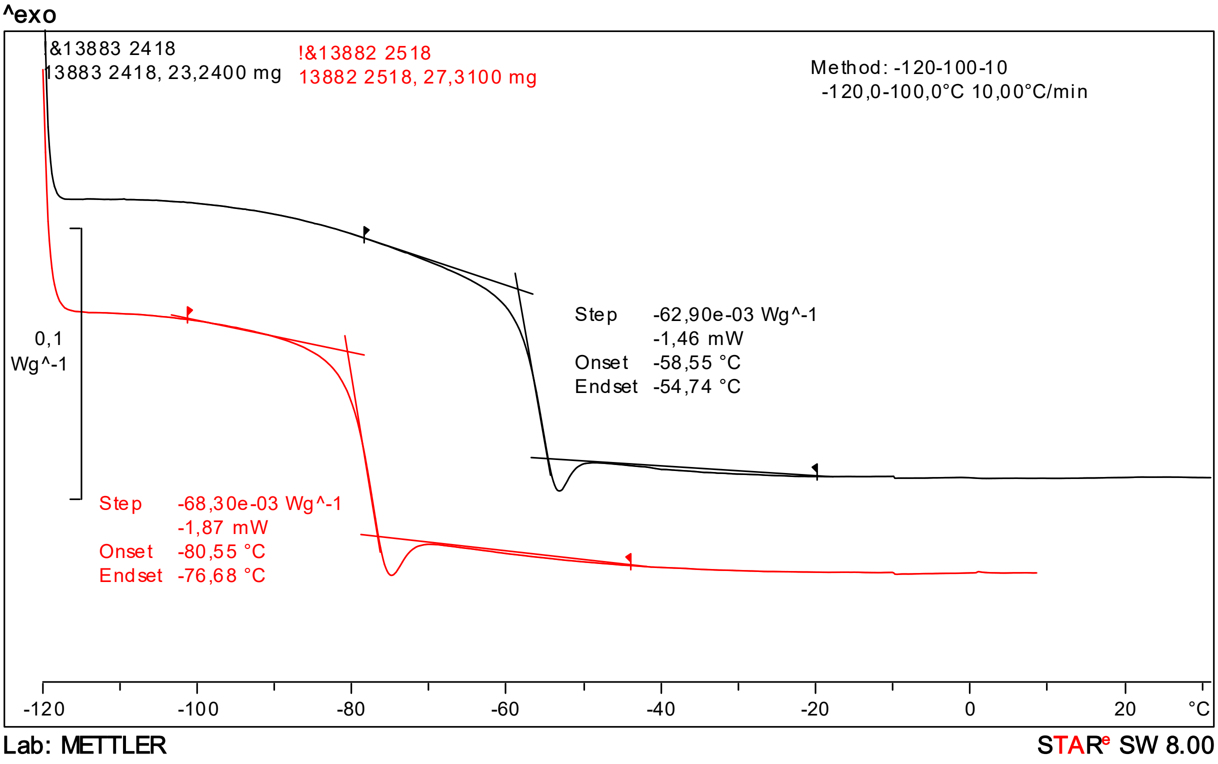

Figure S22. DSC curve of IL 8 (black).
